# Carboxymethyl Cellulose-Based Films for Sustainable Food Packaging: Modification Strategies and Structure–Property Relationships

**DOI:** 10.3390/polym18050552

**Published:** 2026-02-25

**Authors:** Valentina Beghetto, Silvia Conca, Domenico Santandrea

**Affiliations:** 1 Dipartimento di Scienze Molecolari e Nanosistemi, Università Ca’ Foscari Venezia, Via Torino 155, 30172 Venezia, Italy; silvia.conca@crossing-srl.com (S.C.); domenico.santandrea@unive.it (D.S.); 2 Dipartimento di Architettura e Disegno Industriale, Università della Campania “Luigi Vanvitelli”, Via San Lorenzo-Abazia di San Lorenzo, 81031 Aversa, Italy; 3 Consorzio Interuniversitario per le Reattività Chimiche e La Catalisi (CIRCC), Via C. Ulpiani 27, 70126 Bari, Italy; 4 Crossing S.r.l., Viale della Repubblica 193/b, 31100 Treviso, Italy

**Keywords:** carboxymethyl cellulose, physical modifications, chemical modifications, polyelectrolyte complexes, composite polymer blends, cross-linking, bio-based packaging, food packaging

## Abstract

The growing environmental impact of petroleum-based plastics has intensified research into sustainable, biodegradable alternatives for food packaging. Among bio-derived polymers, carboxymethyl cellulose (CMC) has attracted increasing attention due to its abundance, non-toxicity, biodegradability, and excellent film-forming ability. Nevertheless, the intrinsic hydrophilicity and limited mechanical strength of neat CMC restrict its direct application in packaging systems. This review provides a comprehensive and critical overview of recent strategies developed between 2015 and 2025 to enhance the performance of CMC-based films for food packaging applications. Emphasis is placed on physical and chemical modification routes, including polymer blending, polyelectrolyte complex formation, incorporation of functional fillers and nanomaterials, and ionic or covalent crosslinking approaches. The influence of these strategies on key functional properties, such as mechanical behavior, water barrier performance, antimicrobial and antioxidant activity, is systematically discussed. Particular attention is given to CMC-rich systems, enabling meaningful comparison across studies. By highlighting structure–property relationships and identifying current limitations, this review aims to provide guidance for the rational design of advanced CMC-based materials as viable, eco-friendly alternatives to conventional plastic packaging.

## 1. Introduction

The increasing need for biodegradable alternatives to petroleum-based plastics is driven by multiple well-documented environmental and public health impacts [1]. In fact, fossil-derived polymers accumulate in the environment and fragment into micro- and nanoplastics [2,3,4] that are now detected across air, land, and sea [5,6,7,8,9,10,11,12] and that can be transported for long distances by the atmosphere, raising concerns about ecological toxicity and human exposure via inhalation and ingestion [13,14,15]. Moreover, the plastics life cycle, from hydrocarbon extraction through polymer manufacture and final disposal, represents a substantial and growing source of greenhouse gas emissions, so reducing reliance on fossil feedstocks can contribute to climate change mitigation [8]. Finally, mismanagement of plastic waste (landfilling, open burning and uncontrolled incineration) releases particulate matter, persistent organic pollutants (including dioxins/furans) and other toxic combustion by-products that degrade air quality and harm the environment and public health [16,17]. All this considered, there is an urgent need for low-impact, biodegradable packaging solutions.

The packaging industry is a major driver of global plastic consumption, representing nearly 40% of total plastic usage, according to the Plastics Industry Association [18]. This includes packaging for food, beverages, consumer products, and industrial goods. Currently, recycling remains limited, with only 9% of plastic waste being recycled globally [19]; therefore, the need to develop eco-friendly and biodegradable alternatives with acceptable performance, supporting both economic growth and environmental protection, is of utmost urgency. Consequently, increasing attention is being paid to bio-based, bio-degradable plastics from renewable resources [20,21,22].

Cellulose is an unbranched natural polymer composed of repeating glucose units (C_6_H_10_O_5_)_n_ [23], considered the most common organic polysaccharide on Earth. Due to its high abundance in nature, it is one of the most studied biopolymers as a potential feedstock for producing bio-based materials [24,25,26,27]. Historically, cellulosic materials found their primary use in the paper and textile industry [28]; however, significant expansion in their applications has been reported in the last decade, including various fields such as biomedicine [29,30,31,32,33,34], packaging [35,36,37,38,39,40], and nanocomposites [41,42,43,44], among others [45,46,47,48,49].

Cellulose is significantly valuable for packaging applications due to its biodegradability, non-toxicity, and biocompatibility [29,50,51,52]. However, its insolubility in water and common solvents limits its direct use in applications like film preparation or functional coatings. Consequently, various cellulose derivatives have been developed over the years to enhance its processability and expand its application, particularly within the packaging sector [53,54,55,56,57].

The presence of hydroxyl groups in cellulose allows for chemical modification and the introduction of functional groups like acids, chlorides, or oxides, improving its properties, increasing solubility and allowing novel materials to be engineered [58,59,60,61].

The most common water-soluble cellulose derivatives are hydroxyethyl cellulose and carboxymethylcellulose (CMC), obtained by alkali-catalyzed etherification with ethylene oxide and chloroacetic acid, respectively [62,63]. Currently, these two derivatives are employed at industrial level in different areas, for example, in the food, pharmaceutical, cosmetics, detergent, and textile industries [64,65,66,67,68,69]. CMC is largely the most used and synthesized cellulose derivate, with an annual production of 658,000 tons [70,71]. Many recently published papers identify a set of properties that make CMC particularly attractive for food packaging applications: widespread industrial availability; solubility; excellent film-forming ability; transparency [72,73,74,75]; food contact safety and biocompatibility [76]; high tunability with plasticizers, proteins, polysaccharides, nanocellulose, nanofillers, metal oxides and essential oils [77]; and ability to form polyelectrolyte complexes [29,72]. The versatility of CMC enables the rational design of mechanically reinforced, barrier-enhanced films, allowing easy formation of polyelectrolyte complexes and ionic networks, providing structural and functional enhancements without necessarily relying on synthetic additives. These characteristics make CMC systems particularly attractive for sustainable packaging applications, where performance, processability, and environmental compatibility must be simultaneously achieved [35]. Interestingly, although CMC does not exhibit significant intrinsic antimicrobial activity, when functional additives such as essential oils, chitosan, or metal nanoparticles are added, CMC-based films show enhanced biological properties, which are attractive characteristics for the preparation of active food packaging [78].

Nonetheless, the intrinsic hydrophilicity of CMC, arising from the presence of abundant hydroxyl and carboxymethyl groups along its polymer backbone, significantly influences its performance in food packaging applications. These polar functional groups promote strong interactions with water molecules, resulting in high moisture absorption, swelling behavior, and high water vapor permeability (WVP). Consequently, neat CMC films often exhibit reduced dimensional stability and mechanical properties, limiting their applications for food packaging applications.

To overcome these limitations, the most frequent approaches used to enhance the performance of CMC-based films and to develop sustainable alternatives to conventional plastic packaging materials [11,64,72,79,80,81,82] rely mainly on polyelectrolyte complex formation [53,70,72,78], nanofiller reinforcement [34,54,83], and ionic or chemical crosslinking [33,38,84]. CMC-based films are also particularly attractive for edible and biodegradable packaging due to their transparency, safety for food contact, and compatibility with bioactive compounds [76].

As described below, polymer blending with starch, chitosan, gelatin, or other biopolymers primarily improves mechanical strength and flexibility [85]. Polyelectrolyte complex formation, especially with chitosan, enhances film cohesion and moisture resistance through electrostatic interactions [86]. The incorporation of nanomaterials such as cellulose nanocrystals or metal oxide nanoparticles contributes to mechanical reinforcement, UV shielding, and antimicrobial activity [87]. Finally, crosslinking strategies reduce water solubility and improve dimensional stability [57].

Although several reviews have been published in recent years addressing the general properties, applications, and future perspectives of CMC and other cellulose derivatives [64,72,76,77], most of them consider CMC within a wider family of biopolymers or focus on edible films and active packaging without a systematic comparison of formulation strategies and their quantitative impact on structure–property relationships. The novelty of the present review lies in its focus on CMC-rich systems (≥50 wt% CMC) for food packaging applications; we critically analyze studies published between 2015 and 2025. By comparatively evaluating physical blending, polyelectrolyte complex formation, nanofiller reinforcement, and covalent/ionic crosslinking approaches, this work goes beyond the previous literature, correlating modification strategies with mechanical strength, barrier performance, and functional activity. Furthermore, comparison between the mechanical properties of commercial fossil- and bio-based films and discussion of key parameters such as molecular weight and degree of substitution provide a structured framework that facilitates meaningful cross-study comparison for possible future industrial applications.

## 2. Methodology

This systematic review was performed in accordance with PRISMA guidelines. A comprehensive literature search was performed in Scopus and Web of Science, targeting peer-reviewed studies (2015–2025) on the preparation, modification, and application of CMC-based films for food packaging using defined keyword combinations related to CMC, starch blends, film formation methods, and functional additives; additional records were identified through manual reference screening. Studies were included if they reported the preparation of CMC films (>50 wt% of CMC) by casting technology and characterization of films, while non-peer-reviewed works and studies with minor CMC content were excluded. Data extracted include polymer composition, processing method (primarily solution casting), additives, functional performance, and CMC molecular characteristics. Since the focus is the production of films for food packaging systems, the key information reported concerns mechanical performance (tensile strength, elongation at break, and Young’s modulus), barrier characteristics (water vapor permeability and oxygen transmission rate), and functional attributes such as antimicrobial and antioxidant activity. Additional parameters considered are optical transparency, UV shielding capacity, moisture sensitivity, and biodegradability, when available.

## 3. Preparation of CMC Films

Over the years, several processing techniques have been employed for the fabrication of CMC-based films, including solution casting, electrospinning, extrusion, coating, and spray-based technologies [29,72,76,85]. Among these methods, solution casting remains by far the most widely adopted approach at laboratory scale due to its simplicity, versatility, and suitability for incorporating additives, fillers, and functional compounds in a controlled manner [27,53,88,89,90,91,92,93,94,95,96,97,98,99,100].

**Figure 1:**
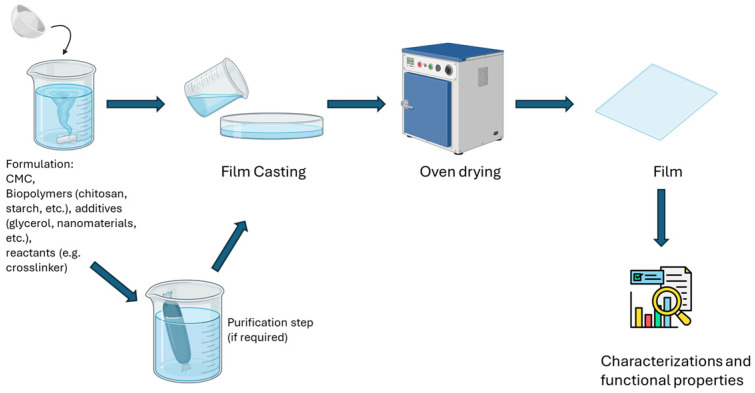
General method for the preparation of CMC films by casting technology.

CMC is dissolved in water with plasticizers (glycerol) and additives, poured into molds, and dried to form coherent films; this method is straightforward and allows easy incorporation of functional agents such as antimicrobials or nanomaterials to tailor film properties [72,101,102] (Figure 1).

Blending CMC with polysaccharides (starch, chitosan) or other biopolymers (also derived from agro-industrial waste) has been extensively investigated in the last 10 years (see below), as an interesting strategy through which to improve tensile properties, water resistance and reduce brittleness while maintaining film-forming ability [103,104,105,106,107,108,109]. The main CMC blends prepared with different polysaccharides or proteins (gelatin) by casting technology and published between 2015 and 2025 are discussed below.

**Figure 2:**
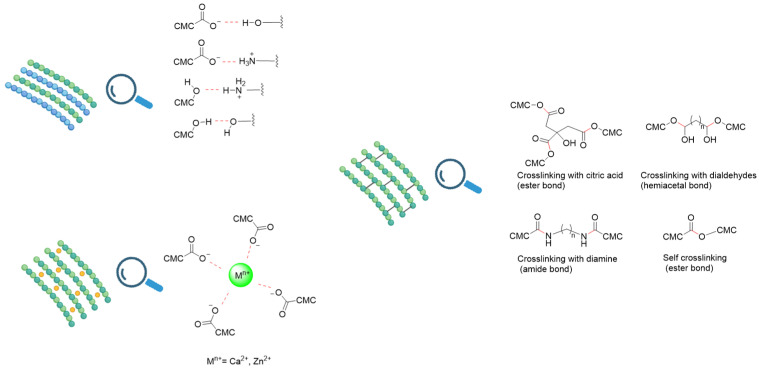
Different strategies for blending and cross-linking of CMC films.

In terms of more industrially relevant processing, thermo-mechanical techniques (extrusion and compression molding) have been developed, in which partially plasticized CMC or CMC blends are processed in the melt, offering higher compatibility with conventional plastic-film lines [76]. Film performance can further be tailored by blending and crosslinking: CMC is combined with starch, proteins, or synthetic biopolymers, and its hydrophilicity is reduced via ionic (Ca^2+^, Zn^2+^), chemical (aldehydes, citric acid), or photo-crosslinking treatments to enhance water resistance and mechanical strength (Figure 2) [110]. Recent reviews on CMC-based and CMC/chitosan (CHI) packaging systems emphasize that the choice and optimization of these preparation techniques—casting vs. thermo-mechanical processing, type and level of plasticizer, emulsion formulation, and crosslinking strategy—are crucial in balancing transparency, strength, barrier properties, and biodegradability for specific food applications [72,78].

Electrospinning has emerged as a promising method with which to produce CMC-based nanofibrous mats with high surface area and tunable porosity, often blended with carriers like polyvinyl alcohol (PVA) or polyvinyl pyrrolidone to overcome processing challenges. These electrospun films can act as breathable, lightweight packaging layers with potential for active food preservation [111], although presently, their application remains limited due to processing complexity and scalability constraints.

Spray drying is not a film-forming method per se, but is used in some advanced formulations for CMC-based materials: solutions or emulsions containing CMC and functional components can be atomized into powders or microcapsules, which may then be further processed into coatings or composite films with controlled release characteristics [112,113]. Finally, coating techniques, including extrusion coating and simpler liquid application methods, are used to deposit thin layers of CMC solutions onto foods or other packaging substrates to form, for example, edible coatings, enhancing barrier performance and shelf life without forming standalone free films [56,114,115,116].

Each of these preparation methods offers distinct advantages and challenges, and the choice among them depends on the desired film morphology, mechanical strength, barrier properties, scalability, and specific food packaging application. Since solution casting emerged as the most common technique used for the preparation of CMC films, the present review will focus on this methodology (Figure 1 and Figure 2).

It should be underlined that although numerous works report the preparation of CMC films by solution casting, enabling tailored control over mechanical, barrier, optical, and antimicrobial properties, direct comparison of reported data is often challenging due to variations in experimental conditions, including CMC molecular weight, degree of substitution, polymer source, and testing protocols, which are not always consistently reported. To address this issue, the present review summarizes key performance parameters, including tensile strength (TS), elongation at break (EB%), water vapor permeability (WVP), moisture content (MC), and water uptake, as reported in the literature (Supplementary Materials section). Where available, CMC molecular weight and degree of substitution are also included to facilitate meaningful comparison. In addition, values obtained for control samples are reported, offering a more comprehensive overview of the effects of formulation and processing strategies on CMC film performance. Finally, to evaluate the potentiality of innovative solutions reported below, physical mechanical data of commercially available polymeric films (both fossil and bio-based) for different food packaging applications are reported in Table 1 [117,118,119].

## 4. Physical and Chemical Modification of CMC

As mentioned above, both physical and chemical modification strategies have been reported in the literature to improve the performance of CMC films obtained by casting technology. In the present sections these two methodologies will be separately discussed.

### 4.1. Physical Modifications

#### 4.1.1. CMC Starch Polyelectrolyte POlymer Blends

Within the many publications present in the literature regarding CMC/corn starch (CS) films, it is important to mention that many of them have CS as the main component, and CMC is only present in minor percentages; therefore, these papers are not reviewed in this work [86,120,121,122,123]. Only polymeric blends prepared with at least 50 wt% of CMC are reported.

Due to the presence of carboxylate groups, CMC is an anionic polyelectrolyte which, in different reaction conditions, can be mixed with a cationic polymer to form a polyelectrolyte complexes (PECs). These physically modified networks, formed by ionic interactions, hydrogen bonding, and chain entanglement rather than covalent bonding, generally outperform single-component films, and properties can be further improved by adding active molecules and nanofillers [72,76,124]. For this reason, physical modification of CMC is particularly attractive for the preparation of films for food packaging applications because it can avoid chemical reagents, reduce regulatory concerns, and preserve the biodegradability and safety of the polymer [77,125].

Different CMC/CS formulations have been studied, and, in many cases, different additives have been used to achieve specific functionalities such as pH responsiveness or antimicrobial activities for the preparation of intelligent or active packaging for food.

Kibar et al., for example, studied composite films based on CS, methylcellulose, and CMC, using glycerol or polyethylene glycol (PEG) as plasticizers [126]. The influence of polymer blend composition and plasticizer on film microstructure, water vapor permeability (WVP), opacity, and solubility was systematically examined. SEM analysis revealed that films plasticized with glycerol possessed a uniform and continuous matrix, indicating good structural cohesion, in contrast to PEG-plasticized films. The WVP values ranged from 1.5 × 10^−11^ to 13.3 × 10^−11^ g/m^2^ Pa.s, and all composite films exhibited improved water resistance compared to CS films. Additional information on the mechanical and WVP properties of CMC/CS films was later reported by Tavares et al. [127]. Tensile strength, EB%, and Young’s modulus (E), together with the WVP of CMC/CS (80/20 *wt*/*wt*) films, reached intermediate values compared to CMC (Table 1) and a modest decrease in thermal properties, due to the lower stability of CMC compared to CS.

In another case, Jiang et al. employed CMC/CS films containing anthocyanins (ATH) extracted from purple sweet potato (PSP) as intelligent packaging to monitor fish freshness [128]. Specifically, these films showed overall good physical mechanical properties (Table 1) and color change from red to blue and green when exposed to different pH or ammonia, meaning they could be used as labels to monitor fish freshness.

Following the work by Jiang, Yun et al. developed intelligent CMC/CS films containing purple sweet potato pigment (PSP) as a redox indicator enabling ready detection of the degree of oil oxidation for household applications [129]. The study reports that CMC/CS films (80/20 *wt*/*wt*) containing 0.25 wt% PSP exhibited the highest sensitivity to oxidized oil, although physical mechanical characteristics were significantly different from other CMC/CS films, showing rather low TS and very high EB%, comparable to BOPP (Table 1). Another example of pH-sensitive films based on CS/CMC/PSP composite films was reported by Silva et al. for the preparation of smart food packaging, yet in this case, the main component of the polymeric blend was CS, and therefore, results are not easily comparable with those reported by Yun [129,130].

**Table 1:** CMC/starch and CMC/CHI composite films.

Composition	MW ^(1)^	DS ^(2)^	TS (MPa) ^(3)^	EB (%) ^(4)^	E (MPa) ^(5)^	Ɵ (°) ^(6)^	WVP ^(7)^	MC (%) ^(8)^	Inh. Zone *E. coli* ^(9)^	Inh. Zone *S. aureus* ^(9)^	Ref.
BOPP ^(10)^	-	-	120	150		102.1	25 ^(11)^				[119]
LDPE ^(12)^	-	-	24	400		80	40 ^(11)^				
PLA ^(13)^	-	-	75	190		81	180 ^(11)^				
Mater Bi ^(14)^	-	-	30	280		90	220 ^(11)^				
Ecovio ^(15)^	-	-	25	500		75	520 ^(11)^				
CMC	-	-	15.80 ± 0.58	11.62 ± 0.63			3.24 × 10^−10^	25.83 ± 0.33	-	-	[105]
CMC	-	-	21.01 ± 2.0	24.96	714.58 ± 25.0	31.25 ± 5.0	8.95 × 10^−10^	27.91 ± 1.81	-	-	[131]
CMC	-	-	50.20 ± 6.90	7.6 ± 2.2	684.3 ± 49.1	53.95 ± 4.76 ^(16)^	-	-	-	-	[127]
CMC	-	-	28.0 ± 2.0	3.0 ± 0.3	1700 ± 50		380 ± 5.0 ^(11)^				[132]
CMC	-	-	40.1 ± 0.9	35.9 ± 1.8	1040 ± 40	39.2 ± 1.8	1.40 × 10^−9^				[133]
CMC	-	-	21.85 ± 3.12	23.42 ± 2.0	385 ± 63		9.2 × 10^−11^				[83]
CMC	-	-	30.83 ± 1.61	7.15 ± 1.5							[134]
CMC	-	-	6.10 ± 0.24	201.73 ± 0.15			0.78 × 10^−10^				[101]
CS	-	-	3.80 ± 0.20	35.1 ± 8.50	47.3 ± 12.5	62.38 ± 3.99 ^(16)^	4.90 × 10^−6^				[127]
CMC80/CS20	-	-	32.60 ± 2.10	21.2 ± 4.3	250.6 ± 2.3	56.73 ± 4.02 ^(16)^	1.57 × 10^−6^	-	-	-	
CMC60/CS40/ATH 0.9 ^(17)^	26,219	0.9	23.69 ± 0.91	14.1 ± 0.55	-	-	-	14.10 ± 0.55	-	-	[128]
CMC80/CS20/PSP 0.25 ^(18)^			2.08 ± 0.06	158.27 ± 0.72				20.67 ± 0.61			[129]
CMC70/CSS30/Q/TBHQ ^(19)^	150	-									[135]
CMC50/CS50/LA 1.5 ^(20)^	-	0.9	4.80 ± 0.56	73.17 ± 2.90	-	-	5.56 × 10^−11^	23.67	-	4.17 ± 0.21	[136]
CMC50/CS50/Men2/Cur2 ^(21)^	250	0.7	36.57 ± 0.10	28.10 ± 0.09		75.30 ± 5.00	2.77 × 10^−11^		17.5	16.5	[15]
CMC70/CHI30/OA50/CEO3 ^(22)^	41	-	3.1 ± 0.4	124.3 ± 9.3		35.6 ± 2.3	0.27 × 10^−7^				[137]
CMC70/CHI30/OA50/GEO3 ^(22)^	41	-	2.7 ± 0.4	61.4 ± 8.8		29.3 ± 1.9	0.99 × 10^−7^				
CMC60/CHI40/EO12 ^(23)^	-	-	5.83 ± 0.30	51.20 ± 6.97	0.12 ± 0.02	119.0		19.21 ± 1.10	7.19		[138]
CMC/HTCC ^(24)^	-	-	21.82	2.27	28.61 ± 0.37	117.0					[139]
CMC90/HTCC10/cry-CaCO_3_ 4 ^(24)^	-	-	35.02 ± 1.68	14.43 ± 0.65	18.72 ± 0.85	68.0 ± 2.6	5.9 × 10^−11^				[140]
CMC/HTCMCh1 ^(25)^	-	-	12.5 ± 2.0	15.0 ± 1.5	20 ± 0.5	70.60 ± 0.61			10.0 ± 0.5	11.0 ± 0.1	[141]

^(1)^ CMC (MW): Molecular weight of CMC in KDa; ^(2)^ DS: degree of substitution of CMC; ^(3)^ TS: Tensile strength (MPa); ^(4)^ EB%: Elongation at break; ^(5)^ E: Young’s modulus (MPa); ^(6)^ Ɵ: Water contact angle; ^(7)^ WVP: Water vapor permeability (g/m^2^ Pa.s); ^(8)^ MC%: Moisture content; ^(9)^ Inh. Zone: Inhibition zone (mm); ^(10)^ BOPP P30: Biaxially oriented coextruded polypropylene film; ^(11)^ WVTR g/m^2^ d; ^(12)^ LDPE: low-density polyethylene film; ^(13)^ PLA: polylactic acid film; ^(14)^ Mater Bi: aromatic/aliphatic biodegradable polyester film; ^(15)^ Ecovio: PolyButyrate Adipate Terephthalate (PBAT) and PLA-based polymeric film; ^(16)^ Water contact angle measured after 60 s; ^(17)^ CMC60/CS40/ATH0.9: Films prepared with CMC/CS 60/40 *wt*/*wt* and 0.9 *wt*/*vol* anthocyanins (ATH); ^(18)^ PSP: Purple sweet potato anthocyanins; ^(19)^ CSS: Cassava starch, Q: quercetin, TBHQ: *tert*-butylhydroquinone; ^(20)^ LA: *Lactococcus lactis*; ^(21)^ Men: *N*-Boc *L*- tryptophan *L*-menthol ester, Cur: Curcumin; ^(22)^ CHI: Chitosan, OA: oleic acid, CEO: cinnamon essential oil, GEO: ginger essential oil; ^(23)^ EO: essential oil; ^(24)^ HTCC: *N*-(2-hydroxyl)-propyl-3-trimethyl ammonium chitosan chloride; ^(25)^ HTCMCh: *N*-2-hydroxylpropyl-3-trimethylammonium-*O*-carboxymethyl chitosan.

Tongdeesoontorn and coworkers additionally studied the properties of CMC/Cassava starch composite films containing 30 wt% glycerol as a plasticizer and variable wt% of quercetin or tert-butylhydroquinone (TBHQ) as an antioxidant [135]. In general, upob increasing the amount of quercetin or TBHQ, a slight decrease in water solubility and EB% were observed, while TS increased compared to control samples (Table 1), probably due to hydrogen bond formation between the additive and the biopolymer chains. These films were tested to reduce the redness of pork meat, observing that the composite films containing quercetin significantly slowed down the decrease in redness of the meat from 50% (control sample) to 25%, slowing the oxidation process of the lard from 35 days to 70 days.

Lan et al. further reported the preparation of CMC/CS antimicrobial films containing Lactococcus lactis as a source of Nisin [136]. The best composite films were obtained with a CMC/CS 50/50 *wt*/*wt* ratio and 1.5% L. lactis, in terms of WVP (5.54 × 10^−11^ g/m^2^ Pa.s) and antimicrobial activity against *Staphylococcus aureus* (53.53%) after 8 days. This work is an interesting example of a viable strategy with which to produce antimicrobial films for food packaging, albeit with modest TS compared to other CMC/CS films (Table 1).

Very recently, Jiang et al. reported the synthesis of two novel esters of L-tryptophan (L-Trp) with L-Menthol or Borneol to develop a fully bio-based and sustainable food packaging with antibacterial and antioxidant properties [15]. A multifunctional composite film was prepared employing CMC/CS (50/50 *wt*/*wt*) in the presence of variable wt% of the synthesized esters and curcumin. Data reported demonstrate that CMC/CS composite films containing 2 wt% of the menthol ester and 2 wt% of curcumin were highly biocompatible with low cytotoxicity and nearly complete antibacterial efficiency against both *Escherichia coli* and *Staphylococcus aureus*. Within the panorama of CMC/CS composites films reported in the literature, albeit with the additional complexity due to the synthesis of the esters, these fully natural material-based films represent a new approach for future green, and renewable bio-based food packaging materials.

Few examples also exist of CMC polymeric blends prepared with carboxylated starch to prepare the film with enhanced mechanical and hydrophobic properties, although generally, starch is the main component of the polymeric blend and therefore beyond the scope of this review [142].

In general, from data reported in Table 1, it emerges that in most cases, the addition of an additive (antioxidant, antimicrobial) not only allows for potentially efficient films for active or intelligent packaging; the overall physical mechanical characteristics are also enhanced. For example, the WVP of CMC/CS (80/20 *wt*/*wt*, 1.57 × 10^−6^ g/m^2^ Pa.s) is five orders of magnitude higher than that reported for CMC50/CS50/1.5LA (5.56 × 10^−11^ g/m^2^ Pa.s) or CMC50/CS50/Men2/Cur2 (2.77 × 10^−11^ g/m^2^ Pa.s), which is of great importance especially for food packaging applications.

#### 4.1.2. CMC Chitosan Polymer Blends

In food packaging applications, CMC/chitosan (CMC/CHI) blends are particularly interesting due to the formation of physically driven PEC, the properties of which strongly depend on pH, charge density, polymer ratio, and ionic strength [78,86]. These variables influence mechanical properties and hydrophobicity, enhancing film cohesion and moisture resistance while also enabling antimicrobial functionality, making such systems suitable for active and edible food packaging [76,115]. A recently published dedicated review on CMC/CHI PEC films highlights how electrostatic interactions underpin improved performance relevant to food packaging [78]. Information on most frequently optimized parameters is given as follows: (i) the CMC/CHI ratio, which is known to control net charge balance and PEC density [141,143,144]; (ii) the influence of pH [145], (iii) crosslinking/ionic strengthening, which reduces water solubility and improve stability [53,146,147,148]; and (iv) the influence of plasticizers and active filler loading materials such as antimicrobials, antioxidants, and nanomaterials [137,138,149,150,151,152].

CMC/CHI composite films exhibit better performance compared to films prepared from a single component. In addition, key characteristics, including mechanical strength, antibacterial effectiveness, and antioxidant capacity, can be significantly improved by incorporating bioactive agents such as essential oils and nanomaterials [153]. Numerous studies have explored the use of CMC/CHI edible films for packaging vegetables, fruits, dry foods, dairy products, and meat products [76], but recent studies most frequently report the use of ionic or covalent crosslinkers to enhance the mechanical properties of CMC/CHI-based films and will be further discussed below [151,154].

Noshirvani et al. examined the influence of cinnamon and ginger essential oils (CEO and GEO) on the properties of CMC/CHI films emulsified with oleic acid (OA) [137]. Water contact angles and EB% raised as oil concentration increased (Table 1), with CEO producing a more pronounced barrier effect (Table 1) and stronger in vitro antifungal activity against Aspergillus Niger compared to those containing GEO. Results indicate that essential oils, especially CEO, can effectively act as plasticizing agents in CMC/CHI films, enhancing moisture barrier performance while retaining antifungal functionality, thereby highlighting their potential for food packaging applications.

Despite the potential of essential oils, limitations in biopolymer-based films due to poor solubility and reduced functional stability have been reported. To address these issues, antibacterial CMC/CHI films have been prepared with nano-emulsions of the essential oil (EON) from Persicaria minor Huds [138]. The best emulsion formulation identified was incorporated into CMC/CS films (1.5/1.0 *wt*/*v*) with EON between 4 and 12% (*v*/*v*). Incorporation of EON gave a very low Young’s modulus of the composite films (0.12 ± 0.02 MPa), indicating improved flexibility (EB% 51.20 ± 6.97%, Table 1). Films also exhibited the most favorable properties in terms of opacity, water solubility (65.5%), and moisture content (19.21%). Additionally, this formulation had strong antibacterial activity against *E. coli* and *Bacillus subtilis*, with inhibition zones of 7.19 and 7.85 mm, respectively.

Few studies have alternatively investigated the use of chitosan-based quaternary ammonium salts in CMC film-forming polymer blends with improved physicochemical characteristics. For example, Wang reported the influence of N-(2-hydroxypropyl)-3-trimethylammonium chitosan chloride (HTCC) on the physicochemical characteristics of CMC films [139]. Film-forming solutions composed of HTCC and CMC at different mass ratios displayed pronounced shear-thinning behavior and typical pseudoplastic flow. Among the formulations, HTCC/CMC films containing 15% HTCC showed the greatest apparent viscosity and the lowest crossover frequency, which can be attributed to the development of strong intermolecular interactions. Although very low EB% values were observed (2.27%), TS values were comparable to those of LDPE (21.82 and 24 MPa, respectively), with one of the highest water contact angles for composite CMC films (117°, Table 1), evidencing the role of HTCC in enhancing CMC-based films’ performance in food packaging.

Based on Wang’s results [139], additional studies were carried out to verify the possibility of improving CMC/HTCC film performance (90/10 *wt*/*wt*) via addition of CaCO_3_ [140]. Calcium carbonate was crystallized in situ (cry-CaCO_3_) within HTCC/CMC film-forming solutions, and its influence on film characteristics, including microstructure, mechanical strength, thermal behavior, optical whiteness, and surface wettability, was systematically evaluated. Films containing commercially available calcium carbonate (com-CaCO_3_) were prepared for comparison. The results revealed that cry-CaCO_3_ facilitated a more uniform dispersion of the CMC/HTCC matrix, improving TS and EB% with reduced hydrophobicity. (Table 1).

Zhang et al. synthesized a zwitterionic chitosan derivative, *N*-2-hydroxylpropyl-3-trimethylammonium-O-carboxymethyl chitosan (HTCMCh), which was incorporated into CMC films to enhance mechanical strength and impart antibacterial functionality [141]. The films were prepared by casting technology in the presence of glycerol as a plasticizer (30 wt% on total biopolymer weight). The presence of 10 wt% HTCMCh with a DS of 0.58 generated the best performing film with an increased contact angle (from 46.20° ± 0.50 to 70.60° ± 0.61), a strong improvement in TS and EB% (+130.9% and +351.6% compared to the control sample, respectively), and delayed bacteria growth on fresh pork surface up to 48 h. These studies clearly demonstrate that the composite design of CMC-based films with other biopolymers, natural extracts, or functional molecules is an effective strategy through which to overcome the intrinsic limitations of pure CMC. Significant improvements can be achieved in terms of mechanical strength, barrier performance, antioxidant, and antimicrobial properties, making these materials more suitable for advanced packaging applications.

#### 4.1.3. CMC Polymer Blends with Other Polysaccharides or Gelatin

Different works have reported on the preparation of binary polyelectrolyte blends made from CMC and various polysaccharides derived from agro-industrial waste. This strategy is becoming increasingly popular as totally biodegradable materials can be produced by recycling waste, thus reducing primary sources depletion and costs [155,156].

For example, Ballesteros et al. studied the incorporation of spent coffee grounds (SCG), which are rich in polysaccharides (arabinose, mannose and galactose), for the preparation, via casting technology, of CMC films [104]. The physical mechanical characteristics of CMC/SCG (90/10 *wt*/*wt*) are similar to those reported for other CMC/CS composites, but an extraordinarily high water contact angle (111°) was achieved, evidencing the high hydrophobic nature of SCG (Table 2).

Another interesting agro-industrial waste is chickpea hulls, an abundant renewable by-product with considerable potential added value. A few examples of CMC films containing polysaccharides derived from chickpea hulls (CHPS) (also in nanocrystal form) have been reported [83,105].

For example, Akhtar studied the incorporation of up to 1 wt% of CHPS with CMC and glycerol (70/30 *wt*/*wt*) [105], showing good TS (31.0 ± 0.56 MPa), and WVP (1.23 × 10^−10^ g/m^2^ Pa.s), together with antioxidant and antibacterial activity against *E. coli* (inhibition zone 10.55 ± 0.69 mm) and *S. aureus* (inhibition zone 13.82 ± 0.44 mm). Additionally, they observed reduced swelling behavior, water solubility, and EB% compared to the CMC control film (Table 1 and Table 2).

Ruan et al. evaluated the performance of composite films based on CMC and sodium alginate (SA) in 50:50 weight ratio [157]. Epigallocatechin gallate (EGCG), extracted from tea plants, was added in different quantities to evaluate its impact on film performance. They found that the addition of 40 wt% of EGCG (the total mass of biopolymers) was able to improve the TS of CMC/SA films from 4.29 ± 0.69 MPa to 10.78 ± 0.15 MPa, although this is rather lower than pristine CMC and commercially available biofilms (Table 1 and Table 2).

Abdollahi et al. studied the preparation of CMC/agar biocomposite films (50/50 *wt*/*wt*) via solvent casting, and the effects of incorporating summer savory essential oil (SSEO, between 0.5 and 1.5% (*v*/*v*)) on the physical mechanical performance and antimicrobial activity of the films [158]. The results showed that SSEO imparted significant antimicrobial activity against *S. aureus*, *Bacillus cereus*, and *Listeria monocytogenes* but lower efficacy against *E. coli*. With 1.0% *v/v* SSEO concentrations, microstructural heterogeneity was observed, leading to good WVP (3.0 × 10^−10^ g/m^2^ Pa.s), although with modest mechanical characteristics (Table 2).

Polysaccharides from mulberry leaves (MLPs) have also been investigated for the development of novel CMC active packaging materials [131]. In China, mulberry leaves are commonly used for various purposes, and therefore, large quantities of residual leaves are available [46]. Akhtar et al. reported the preparation of CMC composite films containing variable wt% of MLP (1, 5, and 10% *w*/*w*). In particular, CMC/MPL films (90/10 *wt*/*wt*) showed substantial reduction in MC% (from 27.91% to 14.12%), WVP (from 8.95 to 5.21 × 10^−10^ g/m^2^ Pa.s) and swelling ratio (from 59.11% to 37.45%) compared to CMC. Also, mechanical performance gradually improved at higher MLP concentrations (Table 1 and Table 2). Additionally, the composite films effectively delay lipid oxidation, highlighting their protective functionality and potentiality as active materials for food packaging applications.

Finally, Spinei et al. reported the formulation of CMC composite films, containing 10 wt% pectin (P), 10 wt% glycerol, and two essential oils, bee bread oil (BBO) and thyme oil (TO) [159]. Incorporation of pectin into CMC-based films led to an improvement in TS compared to CMC (from 56.17 to 65.39 MPa, respectively), which further increased to 70.06 MPa when 3 wt% of TO was added, while simultaneously lowering barrier properties (Table 1 and Table 2). Notably, films containing 2% and 3% BBO or TO underwent complete biodegradation within 20 days of soil burial, demonstrating the promising characteristics of CMC/P/TO films for food packaging applications.

Moreover, polysaccharide gelatin is widely used in film production because it combines excellent functional performance with biocompatibility and sustainability, making it especially attractive for food, pharmaceutical, and biomedical applications [161,162].

In this context, CMC/gelatin (70/30 *wt*/*wt*) films containing 5 wt% CaCl_2_ were developed by He et al., and the performance of films dried in different conditions was compared (between 2 °C and 23 °C) [114]. Data evidence that variations in drying conditions significantly influenced the size and distribution of surface pores, which in turn affected the films’ mechanical strength and WVP. The best WVP and TS values (1.27 ± 0.07 × 10^−10^ g/m^2^ sPa and 53.91 ± 1.69 MPa) were obtained for composites prepared at the lowest temperatures, suggesting that slow evaporation of water generates a stronger network between polymer chains.

#### 4.1.4. CMC Ternary Polymer Blends

Ternary films containing three different biopolymers have also been studied, yet generally, CMC is added only in moderate to very low quantities (<20 wt%) [163,164,165,166]. To the best of our knowledge, only two examples of ternary polymer blends have been published in the last decade, containing over 50 wt% of CMC.

In particular, Lan and coworkers studied the formulation of ternary composite films based on CMC, SA, CHI, and CaCl_2_ [160]. A SA/CHI film-forming solution was added to an aqueous CMC solution, casted, and dried. Then, films containing 85 wt% of CMC and 15 wt% of the SA/CHI mixture (50/50 *wt*/*wt*) were immersed in a CaCl_2_ solution to induce ionic cross-linking, followed by air-drying to obtain CMC/SA/CHI composite films with high TS and enhanced EB% and WVTR compared to CMC (Table 1 and Table 2). Additionally, chitosan imparted effective antimicrobial activity against *E. coli* against *S. aureus* (95.7 ± 5.4% and 93.4 ± 4.7%, respectively).

Functional packaging films based on SA, CMC, and potato starch (PS) were also prepared by Ramakrishnan et al. using a solvent casting approach [80]. Film formulations were prepared by varying the relative proportions of SA/CMC and PS. In one case, SA/CMC/PS in a 1/1/1 wt% ratio was tested, showing good mechanical characteristics (barrier performance against UV radiation, oxygen, and WVP) while simultaneously reducing moisture content and film extensibility (Table 2). Films containing PS exhibited homogeneous thickness, improved thermal resistance, and increased TS.

#### 4.1.5. CMC Polymer Blends Containing Nanopolymer Compounds

Another very interesting and widely explored approach involves the combination of CMC or CMC polymer blends with nanocrystalline cellulose (NC), nanofiber form (NF) or nanochitosan (NCHI) to improve several key aspects of composite films, such as UV and gas barrier, physical–mechanical properties, and, depending on the nature of the nanomaterial, antimicrobial activity [29,72].

**Figure 3:**
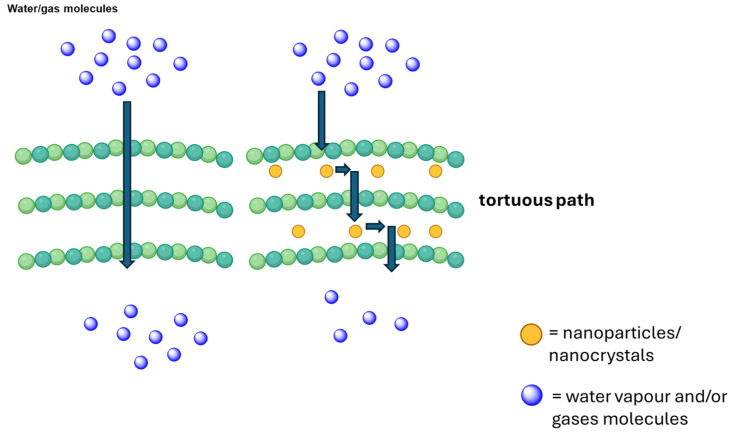
Variations in the performance of films containing nanoparticles or nanocrystals.

Nanomaterials play a critical role in improving the performance of CMC-based films by reinforcing the polymer matrix and introducing active functionalities. Due to their high surface area and strong interfacial interactions with hydroxyl groups of CMC, nanofillers such as cellulose nanocrystals (CNCs), cellulose nanofibers (CNFs), nano-clays, and metal oxide nanoparticles may significantly enhance tensile strength and Young’s modulus by promoting stress transfer and restricting polymer chain mobility (Figure 3). For example, low loadings (typically <5 wt%) of CNCs or CNFs have been widely reported to improve mechanical strength while maintaining transparency, whereas layered nano-clays create a tortuous path that reduces water vapor permeability and oxygen transmission.

Metal oxide nanoparticles such as ZnO and TiO_2_ further provide UV shielding and antimicrobial activity, extending food shelf life, while silver nanoparticles impart strong antibacterial properties at relatively low concentrations. However, the addition of nanoparticles should be carefully studied, since high concentrations may lead to the formation of agglomerates that make the film heterogeneous, with a consequent decrease in overall performance [83,101].

Therefore, optimized loading levels and proper dispersion strategies are essential to balance reinforcement, functionality, and processability in CMC-based nanocomposite films for food packaging applications.

For example, Mandal et al. prepared CMC films reinforced with nanocellulose (NC) from sugarcane bagasse by solvent casting, and the mechanical characteristics, thermal stability, morphology, and WVTR were determined [132]. At optimal NC loading (CMC/NC 50/50 *wt*/*wt*), a substantial enhancement in TS compared to neat CMC was achieved (TS 42.5 ± 1.5 MPa and 28.0 ± 1.5 MPa, respectively), although with very low EB% (1.3 ± 0.5%). Additionally, very transparent composite films were obtained, exhibiting improved thermal resistance and superior WVP properties (Table 3). Later studies have shown that controlled spray-drying conditions can highly improve the EB% of the CMC/NC films [112] (Table 3).

Wei et al. studied CMC composites prepared in combination with tree-like CMC nanofibrils (CMC-NFs) and rod-shaped CMC nanocrystals (CMC-NCs) and the reinforcing effects of these nanofillers in CMC-based composite films [134]. CMC films containing 4 wt% CMC-NFs showed higher TS compared to CMC-NCs and pristine CMC (Table 3), although with very low EB%. In another study, between 1 and 10 wt% of crystalline cellulose nanofibrils extracted from cotton linter pulp (CL-CNFs) were employed as reinforcing fillers in CMC films [133]. Incorporation of 5 wt% CL-CNF led to increases in TS from 40.1 ± 0.9 MPa for CMC to 81.5 ± 3.6 MPa, accompanied by a modest reduction in EB% compared to CMC (Table 1 and Table 3). Further improvement in CMC/CL-CNF films was later reported by Oun and coworkers using ammonium persulfate and grapefruit seed extract as antimicrobial agents (Table 3) [167,168].

Another example of the exploitation of chickpea hull waste has been reported by Li et al. for the preparation of chickpea hull cellulose nanocrystals (CHPS-NCs, between 1 wt% and 10 wt%) and their incorporation in CMC films as reinforcement agents [83]. With CHPS-NCs contents ≤5 wt%, SEM analysis revealed its uniform dispersion within the CMC matrix, resulting in homogeneous film structures. Incorporation of 5 wt% CHPS-NCs significantly enhanced the UV barrier capacity, TS (32.95 ± 4.06 MPa), WVP (4.2 × 10^−11^ g/m^2^ Pa.s), and thermal stability of the films compared with neat CMC films (Table 3). Higher wt% of CHPS-NC, however, lead to an increase in the WVP and lower mechanical properties (TS 21.85 ± 3.12), probably as a consequence of the formation of CHPS-NC agglomerates that reduce the homogeneity of the films.

Jannatyha et al. reported the use of nanochitosan (NCHI) and nanocellulose (NC), into CMC film-forming solutions at concentrations of 0.1, 0.5, and 1 wt% together with 50 wt% glycerol [101]. The incorporation of both NCHI and NC significantly reduced the water solubility and moisture absorption of the CMC films, with the most pronounced effects observed with 1 wt% nanoparticle loading (see Table 3). These reductions were significantly greater in CMC/NCHI films compared to CMC/NC films, while WVP decreased as the nanofiller content increased in both nanocomposite systems. In addition, TS and EB% improved with increasing nanofiller concentration; however, higher nanofiller loading (>1%) resulted in partial nanoparticle aggregation within the CMC matrix. The antibacterial activity of the NCHI films was evaluated against five pathogenic microorganisms, showing strong inhibitory effects (Table 3).

Recently, Mohammadi et al. employed response surface methodology (RSM) to evaluate how different wt% of CMC (0.75–1.75 wt%), Commiphora mukul polysaccharide (CMP, 0–1 wt%) and chitosan nanofibers (CHI-NF, 0–1 wt%) influence the physical and antimicrobial performance of CMC-based nanocomposite films [171]. The optimization strategy focused on maximizing TS, EB%, and antibacterial efficiency, while simultaneously reducing WVP, solubility, swelling behavior, moisture content, opacity, and total color difference. The results showed that the addition of both CMP and CHI-NF contributed to lower moisture content and WVP as well as improved tensile strength. In contrast, increasing the concentrations of CMP and CHI-NF produced mixed effects on EB%, color difference, and swelling capacity. CMP incorporation led to higher opacity and solubility, whereas CHI-NF addition decreased these parameters. Using the RSM-based optimization approach, the formulation comprising 1.5 wt% CMC, 0.25 wt% CMP, and 0.75 wt% CHI-NF was identified as optimal, exhibiting balanced physical, mechanical, and antimicrobial properties.

El Miri et al. focused on the preparation of environmentally friendly bio-nanocomposite films based on CMC/CS blends (70/30 *wt*/*wt*) reinforced with sugarcane bagasse cellulose nanocrystals (SB-NCs) at varying concentrations ranging from 0.5 to 5.0 wt% [169]. The authors deeply studied the rheological behavior of the films such as steady shear viscosity and dynamic viscoelastic properties, demonstrating that the presence of SB-NCs allowed film-forming formulations processable at room temperature to be obtained. With SB-NCs content up to 2.5%, a noticeable reduction in WVP was observed, along with gradual improvements in EB% and TS (Table 3). Overall, this study outlines an effective approach for producing sustainable bio-nanocomposite films by combining CMC, CS, and NC, yielding materials with enhanced transparency, moisture barrier performance, and mechanical strength suitable for packaging applications.

Additionally, multifunctional smart films based on CMC and agar blends were developed by Roy et al., incorporating cellulose nanocrystals (NC) extracted from onion peel (OP) along with shikonin (ShK), a traditional Chinese medicine extracted from the roots of Lithospermum erythrorhizon [124]. The incorporation of OP-NC markedly enhanced the TS, water resistance, and optical performance of the films (Table 3). Meanwhile, the presence of shikonin imparted additional functionalities, such as pH-sensitive color responsiveness as well as antioxidant and antimicrobial activities, without noticeably affecting WVP or thermal stability. In addition, films were highly transparent, with improved UV light shielding and strong antioxidant and antibacterial activity. Owing to the combination of improved structural and functional properties, these smart films show interesting performance for active food packaging and quality-monitoring applications.

Amaregouda et al. reported new biocomposite films based on CMC and CS (70/30 *wt*/*wt*) reinforced with chitosan nanoparticles (CHI-NPs) [110]. The CHI-NPs were synthesized through an ionic gelation process involving CHI and SA and subsequently incorporated as a reinforcing component in CMC/CS matrices. A series of CMC/CS/CHI-NP bio-nanocomposite films were prepared with various nanoparticle loadings (between 1 wt% and 9 wt%). The incorporation of CHI-NPs led to marked enhancements in TS compared to CMC/CS films (Table 3). Practical application studies further revealed that these films were effective in prolonging the shelf life of chicken meat by up to 56 h, indicating their potential for active food packaging systems.

Another example of CMC/gelatin-based bio-nanocomposites has been studied [170]. CMC was combined with gelatin-modified montmorillonite (GMMT) as a nanofiller, anthocyanins (ATH) extracted from red cabbage as a pH-responsive color indicator, and Pistacia leaf extract (PLE) as a bioactive component. The incorporation of ATH and PLE resulted in noticeable color changes and decreased film transparency, while significantly enhancing UV shielding (up to 98%). Increasing the PLE concentration led to denser film structures and rougher surfaces, accompanied by reductions in moisture content (from 15.10% to 12.33%), swelling capacity (from 354.55% to 264.58%), surface wettability (contact angle increasing from 80.1° to 92.49°), WVP (from 7.37 to 5.69 × 10^−10^ g/m^2^ Pa.s), and mechanical values without compromising thermal stability (Table 2). Films containing ATH exhibited distinct pH-dependent color responses, with enhanced pigment stability attributed to the formation of ATH–Al^3+^ complexes. The addition of PE imparted strong antioxidant activity, as well as pronounced antimicrobial effects, particularly in films with higher PLE content (1.5 wt%). Despite its complexity, the developed CMC/gelatin-based bio-nanocomposite films, combining antioxidant and antimicrobial functions with pH-sensitive colorimetric behavior, show interesting potential for advanced food packaging applications.

#### 4.1.6. CMC Polymer Blends Containing Metal Nanoparticles

Metallic nanoparticles are also widely employed in the preparation of CMC composites to improve physical–mechanical and antimicrobial properties. The approach involves the preparation of pure CMC or CMC blend solutions and the incorporation of metal nanoparticles before film casting [172,173].

Noshirvani et al. studied active nanocomposite films composed of CMC, chitosan, and oleic acid (CMC/CHI/OA) containing variable wt% of ZnO-NPs (0.5 to 2 wt%) [174]. The incorporation of ZnO-NPs resulted in a reduction in TS, and thermal stability, while simultaneously increasing EB% and surface hydrophobicity (Table 4). In addition, UV light transmission at 280 nm decreased markedly from 17.3% in the control film to 0.2%, 0.1%, and 0.1% in films containing 0.5, 1, and 2 wt% ZnO-NPs, respectively, demonstrating enhanced UV-shielding capability and strong inhibitory effects against Aspergillus niger for film containing 2 wt% ZnO-NPs. These results indicate that CMC/CH/OA/ZnO-NPs films are efficient materials for antimicrobial and UV-protective applications, although the very low TS may limit their use. Based on Noshirvani’s work, Youssef et al. studied a more simplified formulation of bio-nanocomposite films for packaging, adding zinc oxide nanoparticles (ZnO-NPs) into a CMC/CHI matrix (50/50 *wt*/*wt*) without the addition of oleic acid [175]. CMC/CHI containing 8 wt% of ZnO-NP showed enhanced TS, and the water contact angle almost doubled compared to CMC/CHI/OA/ZnO-NP blends (Table 4). Moreover, the films exhibited strong antimicrobial activity against *S. aureus*, *P. aeruginosa* and *E. coli*., effectively extending the shelf life of soft white cheese and demonstrating their potential suitability for food packaging applications.

Additionally, Helmiyati et al. study aimed to evaluate the influence of ZnO–NPs on the physicochemical characteristics of CMC/PVA films (50/50 *wt*/*wt*) and 25 wt% glycerol [176]. Incorporation of 0.5 wt% ZnO-NPs enhanced the mechanical performance of the films, increasing TS from 15.80 MPa for CMC/PVA (50/50 *wt*/*wt*) to 35.50 MPa and EB% from 80% to 220%. The films exhibited effective antibacterial activity against both *E. coli* and *S. aureus*, with inhibition zones of 0.85 ± 0.22 mm and 1.45 ± 0.27 mm, respectively (Table 4). Overall, CMC/PVA/ZnO-NP films show strong potential as sustainable and environmentally friendly materials for food packaging applications, since due to the presence of PVA and glycerol, these films have very similar mechanical characteristics to the commercially available polymers reported in Table 1.

Another example of CMC/PVA blends containing variable percentages of TiO_2_-NP and a silicone microemulsion (SiME) has recently been reported by Hasanin et al. [177]. The optimal CMC/PVA blending ratio (50/50 *wt*/*wt*) containing 1 wt% TiO_2_-NPs and 5 wt% SiME improved film structure and mechanical performance compared to CMC/PVA films (50/50 *wt*/*wt*), while the water contact angle decreased with increasing quantities of SiME (Table 4). Minimum inhibitory concentration (MIC) values were determined to be 3.125 mg/mL for different bacterial strains, 6.25 mg/mL for unicellular fungi, and 12.5 mg/mL for filamentous fungi.

Li et al. developed multifunctional active films based on CMC/CS (80/20 *wt*/*wt*), ZnO-NPs, 0–5 wt% with or without anthocyanins (ATH) [106]. The inclusion of ZnO-NPs significantly enhanced film flexibility and resistance to water. In addition, increasing ZnO-NPs content changed the properties of the surface from hydrophilic to hydrophobic, with the water contact angle rising markedly from 63.44° to 114.22°. Antibacterial tests revealed that 1 wt% ZnO-NPs was sufficient to achieve effective suppression of *E. coli* and *S. aureus*. Films formulated with both anthocyanins and ZnO-NPs additionally exhibited strong pH responsiveness across a wide pH range (2–11), an important characteristic for active packaging. Overall, an optimized formulation consisting of CMC/CS 80/20 *wt*/*wt* ratio, 3% ZnO-NPs, and 0.1 g ATH was identified as a promising candidate for active food packaging applications (Table 4).

More recently, Kyong and coworkers reported the preparation of novel composite films using CMC and potato starch (PS) reinforced with tellurium nanoparticles (Te-NPs) extracted from banana peel extracts, with strong antibacterial and antioxidant activities [108]. Film prepared with CMC/PS (80/20 *wt*/*wt*) and the addition of 1 mg of Te-NPs and undecanoic acid showed high water vapor barrier properties (WVTR 374.20 ± 2.80 g/m^2^·d), mechanical characteristics (TS 14.85 ± 0.32 MPa, EB% 12.45 ± 3.85%), and high efficiency against *E. coli* and *S. aureus* (Table 4).

### 4.2. Chemical Modifications

Crosslinking or grafting is a common strategy through which to improve CMC performance by forming covalent or ionic bonds between polymer chains, resulting in increased stability, reduced solubility, and improved mechanical integrity [178,179,180]. This modification expands the potential of CMC-based materials for sustainable and functional packaging solutions [181,182,183,184]. Different strategies can be adopted for the chemical modification of CMC, in particular, direct crosslinking of CMC in the presence of a condensation agent, an aldehyde (CA, glutaraldehyde), or in combination with different polymers, as described below [57,185,186,187,188,189].

In general, covalent and ionic crosslinking agents modify the barrier properties of CMC-based films primarily by altering network density, chain mobility, and free volume within the polymer matrix. In covalent crosslinking (using citric acid, glutaraldehyde, or epichlorohydrin), permanent chemical bonds are formed between CMC chains, creating a tighter three-dimensional network that reduces hydrophilicity, limits chain swelling, and decreases WVP and gas permeability. Ionic crosslinking, often achieved through multivalent cations such as Ca^2+^ or Al^3+^, relies on electrostatic interactions between carboxylate groups of CMC and the crosslinking ions. This type of crosslinking forms reversible ionic bridges that increase the density of the polymeric matrix, reducing free volume, thereby improving moisture resistance and dimensional stability [76]. However, excessive crosslink density, whether covalent or ionic, can increase stiffness and reduce flexibility, potentially leading to microcracks that negatively affect barrier performance [57]. Therefore, the improvement in barrier properties depends on achieving an optimal crosslink density that enhances network compactness without compromising film integrity.

#### 4.2.1. Direct Crosslinking of CMC

**Figure 4:**
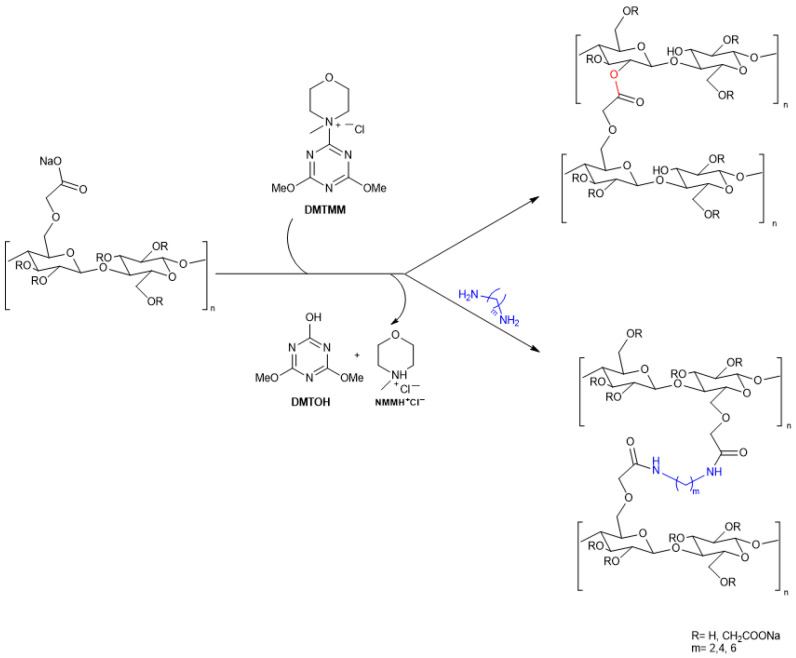
CMC crosslinking in the presence of DMTMM: self-condensation (red functional groups above) and in the presence of a diamine (blue molecule below). Reproduced from [56], RCS Advances 2025.

An interesting approach has been reported in the literature for the self-crosslinking of the carboxylate and hydroxyl groups present on the CMC backbone through an esterification reaction mediated by 4-(4,6-dimethoxy-1,3,5-triazin-2-yl)-4-methylmorpholinium chloride (DMTMM) [57]. DMTMM is a quaternary ammonium salt originally developed for peptide synthesis [190,191,192], collagen crosslinking [193,194,195,196], and amine grafting on hyaluronan [197,198], which has also been successfully employed for the crosslinking of CMC [57]. It enables esterification by forming an intermediate active ester by reacting with the carboxylate groups, which then readily undergoes nucleophilic substitution with an alcohol or an amine. Importantly, the by-products of this reaction, 2,4-dimethoxy-6-hydroxy-1,3,5-triazine (DMTOH) and N-methyl morpholinium hydrochloride (NMM-HCl), are non-toxic and easily removed from the system (Figure 4) [147,197,199]. CMC crosslinked films were prepared by solution casting at 40 °C using DMTMM (2.5 to 10 wt%) and glycerol (25 to 100 wt%) as a plasticizer. The addition of 10 wt% DMTMM and 50 wt% glycerol significantly enhanced the hydrophobic character of the films due to the formation of a compact crosslinked network, leading to a 67% reduction in moisture uptake (MU%) and a 40% decrease in WVP, yielding films that were both flexible and functionally enhanced (Table 5).

Building on these results, further research [56] explored DMTMM-mediated crosslinking in the presence of alkyl diamines of varying lengths (2, 4, and 6 methylene groups) to evaluate the impact of amine-based crosslinkers and amide bond formation on the final properties of CMC films.

These studies were designed to assess how varying the diamine chain length and the molar ratios between the CMC carboxyl groups, DMTMM, and the diamines influenced the degree of crosslinking and, consequently, the film’s performance. The results highlighted that the covalent incorporation of diamines significantly improved mechanical resistance and lowered MC%. This was rationalized by considering that diamines promote more efficient bridging between different polymer chains, resulting in a denser and more interconnected network. Furthermore, the presence of aliphatic chains within the diamine structure likely contributes to more favorable molecular packing and increased hydrophobicity, ultimately reinforcing the overall physical–mechanical performance and reducing the water sensitivity of the material.

**Figure 5:**
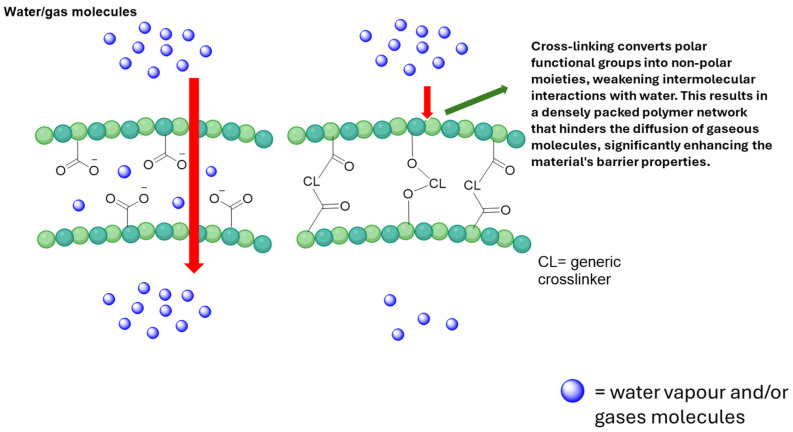
Generic crosslinking mechanism and effects of CMC.

Alternatively, citric acid (CA) is a widely employed crosslinking agent, since it is a bio-based, non-toxic, biodegradable tricarboxylic acid which can form covalent ester bonds with the hydroxyl groups of CMC (Figure 5). The main differences in DMTMM crosslinked CMC are that CA is retained within the CMC structure, and higher temperatures and reaction times are generally required. Recently, Nongnual et al. prepared CA crosslinked CMC films to preserve bananas. Films were prepared in the presence of glycerol as a plasticizer and CA (between 5 wt% and 20 wt%) [185]. The crosslinking reaction was carried out for 10 min at 110 °C followed by one hour at 60 °C and overnight at 50 °C to obtain films by casting technology. The films exhibited a gradual increase in opacity at higher CA loading and a very strong reduction in water uptake. In fact, non-crosslinked CMC films exhibited a very high WU% (about 4000%) after thirty minutes of water immersion, while crosslinked films showed WU% between 40% and 60% after 24 h water immersion. Morais et al. also studied the impact of increasing amounts of CA (from 10 wt% to 30 wt%) on the hydrophobicity of CMC and guar gum (GG) films [200]. The crosslinking reaction was carried out at 140 °C for 30 min. The authors observed a decrease in WVTR from 400 g/m^2^ day for CMC to 150 g/m^2^ day for CMC crosslinked with 30 wt% CA (Figure 5). Unfortunately, both Nongnual and Morais did not report any mechanical characteristics of the films, and therefore, a comparison with other results reported in the literature is difficult to make.

Wang developed CMC-based films crosslinked through a strong bonding network obtained by adding H_2_SO_4_ to an aqueous solution of CMC [186]. The crosslinked films exhibited enhanced water contact angle, while water uptake (WU%) was significantly reduced, with the control film dissolving completely in water after 24 h, whereas acid-treated CMC retained its integrity with only 25% water uptake (Table 5).

Further studies by Santos et al. [201] report the formulation of CMC/CS films (50/50 wt/wt) crosslinked with different concentrations of CA (between 5 wt% and 20 wt%) and 15 wt% glycerol. The addition of CA to the CMC/CS films reduced water swelling, moisture absorption, and WVP, which is one order of magnitude lower than the control sample (CMC50/CS50/Gly15). Although TS is rather low and not significantly different from CMC50/CS50/Gly15 films (7.36 and 6.19 MPa, respectively), a good enhancement in EB% was obtained (Table 5). Thus, these results demonstrate that the CA crosslinking of CMC/CS films significantly influences EB% and WVP.

#### 4.2.2. Crosslinked Polymer Blends

An alternative strategy that has been widely employed is the crosslinking of CMC with other polymers, promoted by a crosslinking agent such as GA, and modified polymers containing aldehydic functional groups, among others [151,187].

For example, Valizadeh et al. evaluated the impact of incorporating cinnamon essential oil (CEO) and oleic acid (OA) in the presence of GA on the antimicrobial, antioxidant, mechanical, physical, and morphological characteristics of CMC/CHI composite films (70/30 *wt*/*wt*) [151]. FT-IR analysis confirmed effective crosslinking induced by GA, which led to a significant enhancement in mechanical strength and a reduction in WVP (Table 5). Crosslinked films containing CEO exhibited antioxidant activity, along with notable in vitro antimicrobial effectiveness against *Listeria monocytogenes* and *Pseudomonas aeruginosa*. The results demonstrate that the incorporation of CEO and GA into CMC/CHI films effectively enhances their antimicrobial, antioxidant, mechanical, and physicochemical performance.

Very recently, Fu and coworkers [187] developed a blend based on CMC, PVA, and dialdehyde cellulose nanofibers CNC (DCNC). Crosslinking takes place thanks to the reaction between the hydroxyl group of polymers and aldehyde groups of DCNC to obtain a compact network. The authors found that the best formulation (60 wt% CMC, 40 wt% PVA, 3 wt% DNCN) showed a good balance between WVP and oxygen permeability barrier (WVP 2.0 × 10^−10^ g/m^2^ Pa.s and OTP 17.0 × 10^−16^ cm^3^·cm/cm^2^·s·Pa), with an improvement in WVP and TS (Table 5). A practical example in strawberry preservation showed a significant improvement of crosslinked samples, which preserved strawberries for up to eight days without significant decay. This data is particularly significant, since previous studies have shown that strawberries packaged with conventional PE film and stored at ambient temperature exhibit pronounced deterioration and decay within four days [205]. Consequently, the application of these films significantly delays spoilage and offers an efficient approach for extending strawberries’ shelf life.

Shan and colleagues developed ternary blend films composed of CMC, PVA, and polyethyleneimine (PEI), crosslinked using GA [188]. In their study, the amounts of CMC, PVA, and GA were kept constant (2:1:1 in weight), while PEI content varied from 0 to 50 wt% compared to the total weight of biocomposite. The crosslinking was supposed to occur through different mechanisms, including acetal–acetal, imine–imine, and imine–acetal bridges. The formulation containing 50 wt% PEI exhibited the most notable improvements in WU%, WVP, TS, UV-shielding capability, and total suppression of *E. coli* and *S. aureus* (Table 5). These enhancements were attributed to the high crosslinking efficiency via Schiff base formation and the intrinsic polycationic nature of PEI, which effectively inhibits bacterial growth.

The oxidation mediated by (2,2,6,6-tetramethylpiperidin-1-yl)oxyl radical (TEMPO) is a very efficient solution for producing oxidized cellulose nanofibers (TEMPO-CNFs) with film-forming capacity for many different applications [117,206,207,208,209,210,211]. TEMPO-CNF can be used for the improvement of CMC films, and Ge et al. studied the development of composite films combining CMC/TEMPO-CNF (90/10 *wt*/*wt*) with 15 wt% glycerol as a plasticizer and 5 wt% or 10 wt% GA as a crosslinking agent, resulting in high TB (112.60 MPa) and consequently modest elasticity (EB% 4.12%, Table 5) [202]. Interestingly, the TB values obtained for CMC/TEMPO-CNF films are high above those of LDPE and other commercially available biopolymers due to the high crosslinking density obtained (Table 1 and Table 5) [205]. To impart active functionality, tea polyphenols (TP) were added (between 2.5 wt% and 15.0 wt%), showing almost total antimicrobial efficacy against *S. aureus* but low efficiency against *E. coli*. The performance of films studied in this work highlights the potential of CMC/TEMPO-CNF-based films as environmentally friendly alternatives to petroleum-derived materials for food packaging applications, although the use of GA and the complexity of the matrix probably make its use at industrial level difficult to adopt.

Additionally, Liang et al. reported the preparation of CMC films in the presence of 1 wt% TiO_2_ and variable quantities of CA and nano-montmorillonite (NMMT) (10 and 20 wt%) [203]. The structure was formed through the self-assembly of CMC and nano-montmorillonite (NMMT), along with the formation of a dual-crosslinked network involving both chemical and physical interactions induced by CA and TiO_2_ nanoparticles. Films prepared with CMC/NMMT (80/20 *wt*/*wt*) containing 1% TiO_2_ exhibited outstanding TS (106.83 MPa), exceeding the performance of most previously reported CMC-based materials and fossil-based polymers (Table 1 and Table 5). In addition to its mechanical strength, the material showed improved water resistance, with water uptake reduced to 42.88%, as well as enhanced thermal stability and UV shielding. The films also demonstrated excellent environmental compatibility, undergoing complete soil degradation within three months.

Sabzevari et al. studied CMC/CS (50/50 *wt*/*wt*) nanocomposite hydrogel films containing 38 wt% glycerol, crosslinked with 25 wt% CA and reinforced with ZnO-NPs (0.5–3 wt%), for the active packaging of fresh broccoli [212]. Films containing 3 wt% ZnO-NP showed the highest WVP (1.11 × 10^−12^ g/m^2^ Pa.s) reported in the literature for CMC polymer blends, although the control sample also had an extraordinary WVP (2.1 × 10^−12^ g/m^2^ Pa.s), which was probably due to the high molecular weight of CMC. Additionally, the contact angle (60.43°), TS, and Young’s modulus were equivalent to CMC/CS films. Antibacterial tests revealed considerable log reductions against *E. coli* (2.41) and *Listeria monocytogenes* (5.69) within 24 h, preserving the freshness and visual appearance of broccoli for 7 days in ambient storage and performing better than traditional polypropylene film packaging. The combination of biodegradability, barrier, mechanical properties, and antimicrobial performance indicates the potential of this CMC/CS/CA/ZnO-NPs system as a promising candidate for application in sustainable active food packaging technology.

Finally, a very interesting work was recently published, reporting the influence of CMC substitution degree (DS) on the physicochemical performance of CMC, potato starch (PS) mixtures (70/30 *wt*/*wt*) crosslinked with CA (15 wt%) [204]. The crosslinked films exhibited a substantial increase in water uptake, accompanied by enhanced rheological behavior. CMC samples with higher DS (1.2) showed superior water absorption, viscosity, and flow properties compared to those with lower substitution levels. Structural characterization further indicated that, although CMC samples with different DS shared similar backbone architectures, higher DS contributed to improved structural stability. However, excessive substitution reduced intermolecular interactions, leading to a partial weakening of the polymer network.

In conclusion, while crosslinking significantly enhances the TS and barrier properties of CMC-based films in most cases, achieving an optimal balance is essential to avoid excessive rigidity. Maintaining sufficient elongation at break remains crucial to ensure the material’s flexibility and practical applicability in packaging.

## 5. Mechanisms of Physical and Chemical Modifications

The enhancement of CMC-based film performance derives from distinct molecular mechanisms associated with physical and chemical modification strategies, and therefore, understanding of these interactions is essential for rational material design. Degree of substitution (DS) and molecular weight primarily control aqueous solubility, solution rheology, and film mechanical integrity. Higher DS increases charge and solubility (raising viscosity and hygroscopicity), whereas higher molecular weight improves tensile strength [213].

Physical modifications do not involve covalent bond formation but rely on intermolecular interactions such as hydrogen bonding, electrostatic attraction, chain entanglement, and filler–matrix interfacial interactions. In polymer blending systems, such as CMC/CS or CMC/CHI, hydrogen bonding between hydroxyl and carboxyl groups promotes improved interchain cohesion, due to electrostatic crosslinking that increases network density, reduces free volume, resulting in enhanced tensile strength and reduced brittleness [72,76,124]. Consequently, PEC-based systems represent one of the most effective physical modification strategies for improving the overall functionality of CMC-based packaging materials. Moreover, physical modification of CMC is particularly attractive for food packaging applications, because it avoids chemical reagents, reduces regulatory concerns, and preserves the biodegradability and safety of the polymer [77,125].

The incorporation of nanofillers (NCs, NCFs, or metal oxide nanoparticles) enhances mechanical strength primarily through stress transfer mechanisms. Strong interfacial hydrogen bonding restricts chain mobility, reinforcing the polymer network [72,76,124]. Additionally, impermeable nanofillers induce a tortuous diffusion pathway for water vapor and gases, thereby reducing permeability. However, as previously reported, excessive filler loading may lead to agglomeration, creating defects that compromise barrier performance. Plasticization represents another physical modification mechanism, where small molecules increase chain mobility and free volume, improving flexibility but often increasing WVP [101,132].

Ionic crosslinking with multivalent cations forms coordination bridges between carboxylate groups, reducing swelling behavior while preserving some flexibility compared to covalent systems. On the other hand, chemical modifications involve covalent bond formation and permanently alter the polymer architecture. Crosslinking reactions, such as esterification with citric acid, amide formation (mediated by a coupling agent), or aldehyde-based Schiff reactions, create three-dimensional networks that restrict polymer chain mobility and reduce free volume within the polymer matrix [57]. This results in enhanced dimensional stability, lower water solubility, and decreased WVP. However, excessive crosslink density or hydrophobic modification may increase stiffness and reduce elongation at break [57,151,187].

Another important feature regards the balancing of mechanical strength and biodegradability in CMC-based films, as strategies that enhance tensile strength, such as high crosslink density or incorporation of inorganic fillers, may reduce biodegradation rates by limiting water penetration and microbial accessibility. To address this trade-off, researchers commonly employ moderate crosslinking levels, bio-based nanofillers, and compatible biodegradable polymer blends, without introducing persistent synthetic components. Additionally, the use of food-grade plasticizers and PEC systems (for example, CMC/CHI) allows for the improvement of mechanical performance while maintaining sufficient hydrophilicity for microbial attack under composting conditions [214]. By carefully controlling formulation parameters such as filler loading, crosslink density, and degree of substitution, it is possible to achieve mechanically robust films that retain acceptable biodegradation behavior, ensuring both functional performance and environmental compatibility [35,214,215].

Overall, the macroscopic properties of CMC-based films are governed by the balance between intermolecular interactions, network compactness, chain mobility, and moisture affinity. Therefore, the selection of modification strategy should be guided by the targeted balance between mechanical strength, barrier performance, flexibility, and processability.

Thus, rational material design requires careful selection of DS, molecular weight, counterion and post-modification (crosslinking, grafting, filler type/dispersion) to meet the competing targets of barrier effectiveness, mechanical toughness, processability and regulatory capacity, and end-of-life constraints for food packaging [216,217].

## 6. Industrial Feasibility and Scale-Up Considerations

Although CMC-based films have demonstrated promising physical mechanical and functional properties at laboratory scale, their adoption at industrial scale for food packaging applications requires systematic evaluation of scalability, processing compatibility, economic viability, and environmental performance. The majority of studies discussed in this review rely on solution casting, a versatile and reproducible laboratory technique that facilitates the incorporation of plasticizers, nanofillers, bioactive agents, and crosslinkers but is of little industrial applicability. In fact, solution casting requires high amounts of water, long drying times, and significant energy consumption associated with solvent evaporation, limiting its direct industrial scalability for high-volume packaging applications.

From an industrial standpoint, thermo-mechanical processing routes such as extrusion and compression molding offer greater compatibility with established plastic film production lines. Nevertheless, the intrinsic hydrophilicity, strong intermolecular hydrogen bonding, and limited thermal stability of CMC create challenges during melt processing [111]. To overcome these issues, blending with thermoplastic polymers such as PVA or the use of high plasticizer content is often required [76]. While these strategies improve processability, they may alter mechanical balance, moisture sensitivity, biodegradability, and regulatory status, necessitating further optimization and compliance verification for food contact applications.

A more pragmatic near-term pathway involves the use of CMC as a functional coating or as a component of multilayer structures applied onto paper or biodegradable polymer substrates. This hybrid strategy minimizes material consumption, reduces drying energy requirements compared to standalone film production, and leverages existing roll-to-roll coating and lamination technologies widely adopted in the packaging sector. In such configurations, CMC layers can provide barrier enhancement, antimicrobial functionality, or intelligent colorimetric responses while maintaining structural support from conventional substrates [52].

Cost considerations remain central to industrial adoption. Although CMC benefits of a large supply chain, high-performance grades with controlled molecular weight and DS may be more expensive than fossil-based polyolefins. The incorporation of nanofillers, essential oils, metal nanoparticles, or advanced crosslinkers further increases formulation complexity and raw material costs, while also requiring dedicated dispersion equipment and stringent quality control. In addition, drying energy, process water management, and food contact migration testing can substantially influence operational expenditure. Therefore, techno-economic analyses comparing CMC-based systems and fossil-based and bio-based packaging materials are essential to assess true market competitiveness.

Environmental and regulatory aspects are equally critical. CMC-based films are governed by the same food contact safety and environmental rules that apply to other packaging materials: manufacturers must use food-grade grades/additives, demonstrate low migration into food, and meet regional food-contact legislation (FDA rules in the US and Regulation (EC) No 1935/2004 in the EU) [218,219]. While CMC is bio-based, biodegradable, and available at food grade, the inclusion of synthetic polymers or inorganic nanoparticles may complicate compostability and recycling pathways. Additionally, the general safety and inertness requirements of the Food Contact Materials framework [219] and any specific implementing measures, including migration testing, should be performed when modifications, the addition of components, and any other transformations are performed. Consequently, future development should prioritize food-grade additives and maintain compatibility with existing organic waste streams where compostability is claimed [220]. Furthermore, comprehensive life cycle assessment studies are required to quantify environmental impacts, including energy consumption during drying, greenhouse gas emissions, water use, and end-of-life scenarios [221,222].

Finally, formulations containing nanoscale fillers or novel active agents require specific risk assessment and clear evidence on migration, toxicology, and end-of-life behavior to satisfy regulators and avoid misleading environmental claims, which is a key reason many commercial CMC applications favor authorized additives and PEC approaches. Regarding the use of nanoparticles in food contact packaging, the primary safety concerns regard particle migration into food, potential toxicological effects after ingestion, and environmental persistence, risks that depend strongly on particle type, size, surface chemistry, concentration, food matrix, and storage conditions. Migration has been documented or modelled for common additives (Ag, ZnO, TiO_2_, clays) particularly under acidic, fatty or high-temperature conditions, so migration testing and material-specific risk assessment are essential [223]. Under certain exposure scenarios, ingested nanoparticles can interact with biological tissues, leading to oxidative stress, inflammation and microbiota damage especially for some materials (notably nano-silver), although toxic effects are dose- and chemistry-dependent [224]. Poorly controlled or aggregated nanoparticles may detach more readily from poorly dispersed composites, increasing exposure risk, while some nanomaterials are bioaccumulative or environmentally persistent after disposal, raising ecological concerns [225]. Regulatory bodies therefore recommend comprehensive, nanoscale-sensitive characterization, standardized migration protocols and targeted toxicological testing (including consideration of particle size/fraction at the nanoscale) before approval, measures recently reinforced in EFSA guidance to ensure consumer safety and enable responsible use in food packaging [226].

To facilitate industrial translation, future research should focus on (i) high-solids and low-energy processing routes to reduce drying costs; (ii) standardized reporting of molecular weight, degree of substitution, and barrier testing protocols to improve cross-study comparability; (iii) pilot-scale validation under realistic humidity and storage conditions; and (iv) integrated techno-economic and LCA studies to support evidence-based material selection.

Overall, CMC-based films represent a credible and environmentally promising alternative for next-generation food packaging, particularly in coating and multilayer configurations in the short term. Broader substitution of conventional plastics will depend on advances in scalable processing, cost reduction strategies, and validated environmental performance, requiring coordinated efforts between academia, material producers, packaging converters, and regulatory authorities.

## 7. Conclusions

Carboxymethyl cellulose has emerged as one of the most versatile and promising bio-based polymers for the development of sustainable food packaging materials. Its renewability, safety, and film-forming capability make CMC an attractive candidate to replace petroleum-derived plastics; however, its inherent water sensitivity and moderate mechanical performance require targeted modification strategies.

This review highlights that significant improvements in CMC film performance can be achieved through polymer blending, polyelectrolyte complex formation, incorporation of functional additives and nanofillers, and chemical or ionic crosslinking. In particular, blending CMC with polysaccharides such as starch, chitosan, alginate, or agro-waste-derived biopolymers effectively enhances mechanical strength, flexibility, and barrier properties while maintaining biodegradability. The formation of polyelectrolyte complexes with cationic polymers, especially chitosan, represents a powerful physical modification route, enabling improved cohesion, moisture resistance, and antimicrobial functionality without compromising environmental compatibility.

The incorporation of nanomaterials, including cellulose nanocrystals, nanofibers, and metal or metal oxide nanoparticles, further expands the functional potential of CMC-based films, leading to enhanced mechanical reinforcement, UV shielding, and active packaging performance. Chemical crosslinking strategies, using both conventional and bio-based crosslinkers, offer substantial gains in water resistance and structural stability, although careful optimization is required to preserve sufficient flexibility for practical use.

It should nevertheless be mentioned that the literature reveals significant inconsistencies and trade-offs that hinder reproducibility and industrial translation. Key gaps that now limit wider adoption include (i) lack of standardized testing protocols and inconsistent reporting (molecular weight, degree of substitution, film thickness, RH/T test conditions), (ii) insufficient pilot-scale/demonstration studies and dispersion/processing solutions for nanofillers, (iii) lack of techno-economic and life cycle analyses comparing CMC systems to fossil-based polymeric films, and (iv) limited long-term ageing, migration and regulatory data for many additive-containing formulations.

Quantitative comparison is often limited by incomplete reporting of key parameters and by substantial variability in raw materials and experimental conditions. Polymer blending with starch, chitosan, or gelatin can improve tensile strength and reduce brittleness, yet performance strongly depends on blend ratio, plasticizer content, and processing conditions, leading to widely divergent barrier properties and mechanical results. Polyelectrolyte complexes offer a chemical-free route to improved cohesion, moisture resistance, and antimicrobial activity, but their effectiveness is highly sensitive to pH, ionic strength, and charge density, complicating reproducibility and scale-up. Nanofillers enhance mechanical strength, UV shielding, and functionality at low loadings, though dispersion challenges and aggregation at higher concentrations can cause embrittlement or reduced barrier performance. Similarly, chemical or ionic crosslinking improves water resistance and dimensional stability but often increases stiffness and reduces elongation, requiring careful optimization to maintain flexibility. Overall, flexibility and water resistance remain a major challenge for CMC systems, governed by chain mobility and network density, in order to achieve application-specific performance and a balanced adjustment of plasticizer content, crosslink density, and filler loading. Standardized characterization and minimum reporting requirements are essential to enable transferable design rules and reliable comparison across studies.

In other words, no single modification strategy is universally optimal, rather, the final film performance strongly depends on polymer composition, degree of substitution, processing conditions, and additive selection. Future research should focus on scalable processing methods, standardized reporting of material parameters, and life cycle assessment to facilitate industrial translation. With continued optimization, CMC-based films represent a realistic and environmentally responsible alternative for next-generation food packaging applications.

## Figures and Tables

**Table 2:** CMC binary and ternary polymer blends with various polysaccharides or gelatin.

Composition	MW ^(1)^	DS ^(2)^	TS (MPa) ^(3)^	EB (%) ^(4)^	E (MPa) ^(5)^	Ɵ (°) ^(6)^	WVP ^(7)^	MC (%) ^(8)^	Inh. Zone *E. coli* ^(9)^	Inh. Zone *S. aureus* ^(9)^	Ref.
CMC90/SCE10 ^(10)^	-	-	22.33 ± 2.35	6.56 ± 0.86		111.48 ± 3.38	3.64 × 10^−10^	20.97 ± 0.82	11.07 ± 0.78	14.25 ± 0.37	[104]
CMC70/Gly30/CHPS 1 ^(11)^	-	-	31.0 ± 0.56	5.96 ± 0.76			1.23 × 10^−10^	20.34 ± 0.69	10.55 ± 0.69	13.82 ± 0.44	[105]
CMC50/SA50 ^(12)^	-	-	4.29 ± 0.69	27.50 ± 2.08							[157]
CMC50/SA50/EGCG 040 ^(13)^	-	-	10.78 ± 0.15		11.20 ± 1.57						
CMC50/Agar50	-	-	5.5	40		42.4 ± 2.4	2.6 × 10^−10^				[158]
CMC50/Agar50/SSEO 1 ^(13)^	-	-	7.5	55		58.3 ± 2.4	3.0 × 10^−10^			33.45 ± 2.76	
CMC90/MLP10 ^(14)^	-	-	34.86	14.23	970	65.45 ± 5.0	5.21 × 10^−10^	14.12 ± 1.70	11.75	9.41	[131]
CMC90/P10/TO3 ^(15)^	-	-	70.06	13.39	533.0		0.34 × 10^−10^				[159]
CMC70/Gelatin30/CaCl^2^ 5	-	-	53.91 ± 1.69	12.26 ± 5.05			1.27 × 10^−10^				[114]
CMC85/SA/CS15	-	-	65.32 ± 14.31	17.85 ± 3.86	8.98 ± 1.51				12.5	10.0	[160]
SA/CMC/PS 1/1/1 ^(16)^	-	-	31.95 ± 0.66	13.78 ± 0.44		62.79 ± 1.00	2.26 × 10^−9^	16.21 ± 0.34			[80]

^(1)^ CMC (MW): Molecular weight of CMC in KDa; ^(2)^ DS: degree of substitution of CMC; ^(3)^ TS: Tensile strength (MPa); ^(4)^ EB%: Elongation at break; ^(5)^ E: Young’s modulus (MPa); ^(6)^ Ɵ: Water contact angle; ^(7)^ WVP: Water vapor permeability (g/m^2^ Pa.s); ^(8)^ MC%: Moisture content; ^(9)^ Inh. Zone: Inhibition zone (mm); ^(10)^ SCE: Polysaccharides extracted from spent coffee; ^(11)^ Gly: glycerol; CHPS: Chickpea hull polysaccharides; ^(12)^ SA: Sodium alginate; EGCG: epigallocatechin gallate; ^(13)^ SSEO: Summer savory essential oil; ^(14)^ MLP: Mulberry leaf polysaccharide; ^(15)^ P: Citrus pectin, TO: Thymol oil; ^(16)^ PS: Potato starch.

**Table 3:** CMC polymer blends containing nanopolymer compounds.

Composition	MW ^(1)^	DS ^(2)^	TS (MPa) ^(3)^	EB (%) ^(4)^	E (MPa) ^(5)^	Ɵ (°) ^(6)^	WVP ^(7)^	MC (%) ^(8)^	Inh. Zone *S. aureus* ^(9)^	Ref.
CMC50/NC50 ^(10)^	-	-	42.5 ± 1.5	1.3 ± 0.5	3750 ± 100		4.32 × 10^−11^			[132]
CMC50/NC50 ^(10)^	80	0.8		34.86 ± 2.90	2308 ± 143	72.0 ± 10.0	6.18 × 10^−11^			[112]
CMC/CMC-NF4 ^(11)^	226	0.8	52.5 ± 2.0	3.5 ± 0.3						[134]
CMC90/CNF10 ^(12)^	-	-	49.2 ± 2.1	26.6 ± 2.2	1330 ± 200	23.9 ± 1.7	1.44 × 10^−9^			[133]
CMC/CL-CNF ^(13)^	-	-	81.5 ± 3.6	22.9 ± 2.1	2637 ± 59	31.5 ± 4.5	1.75 × 10^−9^			[167]
CMC70/Gly30/CHI-NC5/GSE ^(14)^	250	0.9	51.0 ± 0.9	14.2 ± 1.2	1900 ± 4		1.36 × 10^−9^			[168]
CMC/CHPS-NC5 ^(15)^	-	-	32.95 ± 4.06	20.32 ± 0.32	1100 ± 76.78		4.20 × 10^−11^			[83]
CMC/NC1	-	-	12.3 ± 0.3	89.53 ± 0.18			0.28 × 10^−10^	22.0 ± 1.3		[101]
CMC/NC0.5/NCHI0.5 ^(16)^	-	-	9.95 ± 0.45	4.96 ± 0.11			0.11 × 10^−10^	15.0 ± 1.0	3.66 ± 0.57	
CMC70/CS30/SB-NC2.5	-	-	99.06 ± 4.95	20.88 ± 0.83	1375.34 ± 68.80		3.50 × 10^−7^			[169]
CMC50/Agar50/OP-NC5/ShK10 ^(17)^	250	0.9	61.7 ± 5.2	8 ± 2	2600 ± 200	50 ± 4	0.81 × 10^−9^			[124]
CMC70/TS30/CHI-NP ^(18)^	700	0.9	81.08	11.3			3.57 × 10^−7^	12.17 ± 0.89		[110]
CMC50/Gelatin50/GMMT3/ATH1/PLE1.5 ^(19)^	700	0.9				99.5 ± 0.5	5.69 × 10^−10^	10.0 ± 0.34		[170]

^(1)^ CMC (MW): Molecular weight of CMC in KDa; ^(2)^ DS: degree of substitution of CMC; ^(3)^ TS: Tensile strength (MPa); ^(4)^ EB%: Elongation at break; ^(5)^ E: Young’s modulus (MPa); ^(6)^ Ɵ: Water contact angle; ^(7)^ WVP: Water vapor permeability (g/m^2^ Pa.s); ^(8)^ MC%: Moisture content; ^(9)^ Inh. Zone: Inhibition zone (mm); ^(10)^ NC: nano-cellulose; ^(11)^ CMC-NF: carboxymethyl cellulose nanofibrils; ^(12)^ CNF: Crystalline cellulose nanofibril; ^(13)^ CL-CNF: Cotton linter crystalline nanocellulose; ^(14)^ Gly: glycerol, CHI-NC: Chitosan nanocrystals, GSE: Grapefruit seed extract; ^(15)^ CHPS-NC: Chickpea hull cellulose nanocrystals; ^(16)^ NCHI: Nanochitosan; ^(17)^ OP-NC: Onion peel nanocrystals, ShK: Shikonin; ^(18)^ TS: Tapioca starch, CHI-NP: Chitosan nanoparticles; ^(19)^ GMMT: Gelatin montmorillonite; PLE: Pistacia leaf extract.

**Table 4:** CMC polymer blends containing metal nanoparticles.

Composition	MW ^(1)^	DS ^(2)^	TS (MPa) ^(3)^	EB (%) ^(4)^	E (MPa) ^(5)^	Ɵ (°) ^(6)^	WVP ^(7)^	MC (%) ^(8)^	Inh. Zone *E. coli* ^(9)^	Inh. Zone *S. aureus* ^(9)^	Ref.
CMC70/CHI30/OA50	50–190	0.7–0.8	7.34 ± 0.8	13.14 ± 5.27	55.9 ± 2.8	22.9 ± 2.3					[174]
CMC70/CHI30/OA50/ZnO-NP1	50–190	0.7–0.8	4.38 ± 0.59	42.37 ± 3.82	30.27 ± 3.84	47.40 ± 1.25	8.27 × 10^−7^				
CMC50/CHI50/ZnO-NP8	420,000	0.7	12.6			95.6			9.0	11.0	[175]
CMC50/PVA50/Gly25	240.2	-	15.80	80.0							[176]
CMC50/PVA50/Gly25/ZnO-NP 0.5	240.2	-	35.50	220.0					0.85 ± 0.22	1.45 ± 0.27	
CMC50/PVA50	-	0.7	25	22	15	61.66 ± 0.57					[177]
CMC50/PVA50/TiO_2_-NP 1/SiME 5 ^(10)^	-	0.7	33	47	30	25.66 ± 0.57					
CMC80/CS20	-	-	20.83 ± 2.00	25.87 ± 3.09		45.50	1.49 × 10^−10^				[106]
CMC80/CS20/ZnO-NP3			14.91 ± 2.50	31.29 ± 2.90	-	87.85 ± 1.43	1.18 × 10^−10^	14.76 ± 0.55	27.92 ± 0.13	25.27 ± 0.26	
CMC80/CS20/ZnO-NP3/ATH 0.1 g			13.19 ± 1.69	32.14 ± 2.01	-	88.52 ± 0.72	9.64 × 10^−11^	13.90 ± 0.63	31.15 ± 0.21	28.56 ± 0.45	
CMC80/PS20 ^(11)^	250	0.9	16.0 ± 1.0	11.0 ± 5.0			410 ^(12)^				[108]
CMC80/PS20/Te-NP1/UDA1 ^(11)^	250	0.9	14.85 ± 0.32	12.45 ± 3.85			374.2 ^(12)^				

^(1)^ CMC (MW): Molecular weight of CMC in KDa; ^(2)^ DS: degree of substitution of CMC; ^(3)^ TS: Tensile strength (MPa); ^(4)^ EB%: Elongation at break; ^(5)^ E: Young’s modulus (MPa); ^(6)^ Ɵ: Water contact angle; ^(7)^ WVP: Water vapor permeability (g/m^2^ Pa.s); ^(8)^ MC%: Moisture content; ^(9)^ Inh. Zone: Inhibition zone (mm); ^(10)^ SiME: Silicone microemulsion; ^(11)^ PS: Potato starch, Te-NP: Tellurium nanoparticles, UDA: Undecanoic acid; ^(12)^ WVTR g/m^2^ d.

**Table 5:** Crosslinked CMC.

Composition	MW ^(1)^	DS ^(2)^	TS (MPa) ^(3)^	EB (%) ^(4)^	E (MPa) ^(5)^	Ɵ (°) ^(6)^	WVP ^(7)^	MC (%) ^(8)^	Inh. Zone *E. coli* ^(9)^	Inh. Zone *S. aureus* ^(9)^	Ref.
CMC/Gly50/DMTMM10 ^(10)^	90	0.7	52.0 ± 3.0	37.0 ± 1.5			1.09 × 10^−7^	45.59 ± 2.01			[57]
CMC/DMTMM1/EDA0.5	90	0.7	75 ± 2.9	4.7 ± 0.6			2.67 ± 0.21 × 10^−10^	38.5 ± 4.3			[56]
CMC/CA20		0.84					320 ^(11)^	20 ± 2			[185]
CMC50/GG50/CA10 ^(12)^	-	-					308 ± 48 ^(11)^				[200]
CMC50/CS50/Gly15	-	0.25	6.19	55.48	13.37		1.05 × 10^−10^	16.00			[201]
CMC50/CS50/Gly15/CA1.3	-	0.25	7.36	77.82	23.32		6.42 × 10^−11^	13.37			
CMC/H_2_ SO_4_ 5	-	-	29.6 ± 5.0			80.0					[186]
CMC70/CHI30/CEO/OA/GA	41	-	7.99 ± 0.97	66.97 ± 3.85			6.23 × 10^−6^				[151]
CMC60/PVA40	-	-	45.0	22.0			3.0 × 10^−10^				[187]
CMC60/PVA40/DCNC 3 ^(13)^	-	-	90.0	7.0			2.0 × 10^−10^				[187]
CMC2/PVA1/GA1/PEI0.5	-	-	65.17	7.12	2800	85	0.3 × 10^−10^				[188]
CMC8/CNF1/Gly/GA5/TP10 ^(14)^	-	-	112.60	4.12		85.25 ± 2.5					[202]
CMC80/NMMT20/CA20/TiO_2_ 1	700	0.9	106.83 ± 5.00	2.6 ± 1.0					31.5 ± 0.50	28.67 ± 1.04	[203]
CMC50/CHI50/CA30/Gly38	150–250	0.55–0.65	7.12	35.95	0.22	45.19	2.10 × 10^−12^				[204]
CMC50/CHI50/CA30/Gly38/ZnO-NP3	150–250	0.55–0.65	7.54	29.27	0.96	60.43	1.11 × 10^−12^				

^(1)^ CMC (MW): Molecular weight of CMC in KDa; ^(2)^ DS: degree of substitution of CMC; ^(3)^ TS: Tensile strength (MPa); ^(4)^ EB%: Elongation at break; ^(5)^ E: Young’s modulus (MPa); ^(6)^ Ɵ: Water contact angle; ^(7)^ WVP: Water vapor permeability (g/m^2^ Pa.s); ^(8)^ MC%: Moisture content; ^(9)^ Inh. Zone: Inhibition zone (mm); ^(10)^ DMTMM: 4-(4,6-dimethoxy-1,3,5-triazin-2-yl)-4-methylmorpholinium chloride; ^(11)^ WVTR g/m^2^ d; ^(12)^ GG: Guar gum; ^(13)^ DCNC: dialdehyde nanocellulose; ^(14)^ GA: Glutaraldehyde; TP: Tea polyphenols; EDA: Ethylene-diamine.

## Data Availability

No new data were created or analyzed in this study. Data sharing is not applicable to this article.

## Supplementary Materials

The following supporting information can be downloaded at: https://www.mdpi.com/article/10.3390/polym18050552/s1. References [227,228] are cited in the supplementary materials. S1. CMC Film and coating characterization. S1.1. Moisture uptake (MU%). S1.2 Water uptake (WU%). S1.3 Water solubility (WS%). S1.4 Water vapor transmission rate (WVTR) and water vapor permeability (WVP). S1.5 Oil resistance ability of films (OAR %). S1.6 Mechanical properties: tensile strength (TS) and elongation at break (EB%). S1.7 Opacity and UV-barrier properties. S1.8 Antioxidant activity. S1.9 Antimicrobial properties.

## Abbreviations

The following abbreviations are used in this manuscript:

ATH	Anthocyanins
BBO	Bee bread oil
CA	Citric acid
CEO	Cinnamon essential oil
CESO	Clove essential oil
CHI	Chitosan
CHI-NF	Chitosan nanofibers
CHI-NP	Chitosan nanoparticles
CHPS	Chickpea hull polysaccharides
CHPS-NCs	Chickpea hull polysaccharide nanocrystals
CL-CNFs	Cotton linter cellulose nanofibrils
CMC	Carboxymethyl cellulose
CMC-NCs	Carboxymethyl cellulose nanocrystals
CMC-NFs	Carboxymethyl cellulose nanofibrils
CMP	*Commiphora mukul* polysaccharide
CNC/CNCs	Cellulose nanocrystal(s)
CNF/CNFs	Cellulose nanofiber(s)
CS	Corn starch
CIEO	Cinnamon essential oil (nano-emulsion form)
DCNC	Dialdehyde cellulose nanofibers
DMTOH	2,4-Dimethoxy-6-hydroxy-1,3,5-triazine
DMTMM	4-(4,6-Dimethoxy-1,3,5-triazin-2-yl)-4-methylmorpholinium chloride
DS	Degree of substitution
EB%	Elongation at break
EGCG	Epigallocatechin gallate
EON	Essential oil nano-emulsion
FT-IR	Fourier transform infrared spectroscopy
GA	Glutaraldehyde
GEO	Ginger essential oil
GG	Guar gum
GMMT	Gelatin-modified montmorillonite
HTCC	*N*-(2-hydroxypropyl)-3-trimethylammonium chitosan chloride
HTCMCh	*N*-2-hydroxylpropyl-3-trimethylammonium-*O*-carboxymethyl chitosan
LA	*Lactococcus lactis*
MC%	Moisture content %
MLP	Mulberry leaf polysaccharides
MS-Q188	Quaternized starch (Q188 grade)
MU%	Moisture uptake
NC	Nanocellulose
NCHI	Nanochitosan
NMMT	Nano-montmorillonite
OA	Oleic acid
OP-NC	Onion peel nanocellulose
OTR	Oxygen transmission rate
PEC	Polyelectrolyte complex
PE	Polyethylene
PEI	Polyethyleneimine
PLE	Pistacia leave extract
PRISMA	Preferred Reporting Items for Systematic Reviews and Meta-Analyses
PS	Potato starch
PSP	Purple sweet potato pigment
PVA	Poly(vinyl alcohol)
RSM	Response surface methodology
SA	Sodium alginate
SB-NC	Sugarcane bagasse cellulose nanocrystals
SCG	Spent coffee grounds
SEM	Scanning electron microscopy
ShK	Shikonin
SSEO	Summer savory essential oil
TBHQ	*tert*-Butylhydroquinone
TEMPO	(2,2,6,6-Tetramethylpiperidin-1-yl)oxyl radical
TEMPO-CNF	TEMPO-oxidized cellulose nanofibers
TO	Thymol oil
TP	Tea polyphenols
TS	Tensile strength
UV	Ultraviolet
WVTR	Water vapor transmission rate
WVP	Water vapor permeability
WU%	Water uptake percentage
ZnO-NPs	Zinc oxide nanoparticles
