# Supplementary material for: Carboxymethyl Cellulose-Based Films for Sustainable Food Packaging: Modification Strategies and Structure–Property Relationships
Source: Polymers (Basel). 2026 Feb 25;18(5):552. doi: 10.3390/polym18050552 (PMC12986814; doi:10.3390/polym18050552)
Supplement: Supplementary file 1 [file polymers-18-00552-s001.zip › polymers-4164969-supplementary.pdf]

SUPPLEMENTARY MATERIAL

Carboxymethyl Cellulose-Based Films for Sustainable Food Packaging: Modification Strategies and Structure–Property Relationships

Valentina Beghetto [1,2,3,4]*, Silvia Conca [1,4] and Domenico Santandrea [1,2,3]

[1] Dipartimento di Scienze Molecolari e Nanosistemi, Università Ca' Foscari Venezia, Via Torino 155, 30172, Venezia, Italy; beghetto@unive.it, domenico.santandrea@unive.it
[2] Dipartimento di Architettura e Disegno Industriale, Università della Campania "Luigi Vanvitelli", Via San Lorenzo-Abazia di San Lorenzo 81031 - Aversa (CE)
[3] Consorzio Interuniversitario per le Reattività Chimiche e La Catalisi (CIRCC), Via C. Ulpiani 27, 70126, Bari, Italy
[4] Crossing S.r.l., Viale della Repubblica 193/b, 31100 Treviso, Italy, valentina.beghetto@crossing-srl.com, silvia.conca@crossing-srl.com
* Correspondence: beghetto@unive.it; Tel.: +39-041-2348928

Index:

S1. CMC Film and coating characterization

S1.1. Moisture uptake (MU%) — *page 2*

S1.2 Water uptake (WU%) — *page 2*

S1.3 Water solubility (WS%) — *page 3*

S1.4 Water vapor transmission rate (WVTR) and water vapor permeability (WVP) — *page 3*

S1.5 Oil resistance ability of films (OAR %) — *page 3*

S1.6 Mechanical properties: tensile strength (TS) and elongation at break (EB%) — *page 4*

S1.7 Opacity and UV-barrier properties — *page 4*

S1.8 Antioxidant activity — *page 5*

S1.9 Antimicrobial properties — *page 5*

S1. CMC Film characterization

Detailed characterization of the films is crucial to understand the performances of films and coatings and to determine possible final applications. This characterization typically involves the evaluation of several key functional properties, such as moisture uptake (MU%) and water uptake (WU%) to assess hydrophilicity, water vapor transmission rate (WVTR) and water vapor permeability (WVP) to evaluate water vapor barrier properties, oil absorption rate (OAR%) to determine the resistance to lipids permeation. Physical-mechanical characteristics are measured by tensile strength (TS) and elongation at break (EB%), while transparency, opacity, and colour, are determined by UV-Vis spectrophotometry and colorimetric analysis. This systematic approach to property assessment provides a robust framework for comparing and optimizing film and coating formulations.
To allow for a better understanding of the reader a brief description of different methodologies listed above is reported.

S1.1. Moisture uptake (MU%): The moisture uptake determines how much moisture the film can absorb from the environment and allow the calculation of the affinity of the material towards moisture through eq. 1:

$$MU\% = \frac{w_{cond} - w_{dry}}{w_{dry}} * 100 \quad (1)$$

where w_{cond} is the weight of the sample conditioned in a given relative humidity (RH%) for a certain period (usually 48 h). Sometime the MU% is reported as a function of conditioning time or as a function of different RH% employed for sample conditioning. RH% of 55%, 75% and 97% are commonly employed. Different terminologies are adopted by different authors for this parameter, like moisture content % (MC%) or moisture absorption % (MA%).

S1.2 Water uptake (WU%)**:** Water uptake is the amount of water absorbed by a sample immersed in water for a specific time and is usually calculated by eq. 2 where w_{wet} is the weight of the sample after a given period water immersion and w_{dry} is the dry weight of the sample.

$$WU\% = \frac{w_{wet} - w_{dry}}{w_{dry}} * 100 \quad (2)$$

As for MU%, when developing coatings for packaging applications the fundamental aim is to reduce WU% to the lowest possible level.

S1.3 Water solubility (WS%): it refers to the ability of the material to retain its starting weight after water immersion, avoiding disintegration and solubilization. The aim is to decrease the WS% as much as possible to achieve high water-resistance of the sample. WS% is calculated by eq. 3.

$$WS\% = \frac{w_t - w_0}{w_0} * 100 \qquad (3)$$

where w_t is the weight of the sample usually after 24 h of immersion in water and w_0 is the starting weight of the sample. To evaluate the effect of a given additive or chemical process carried out on CMC, the comparison between control sample and tested sample will be given for each work reported in this review.

S1.4 Water vapor transmission rate (WVTR) and water vapor permeability (WVP): WVTR and WVP are very important parameters related to moisture barrier properties of films potentially employable in packaging. The typical experimental procedure involves the use of a cup filled with an anhydrous substance (usually calcium chloride or silica gel), sealed with the sample on the top. The whole system is then placed in a conditioned atmosphere with specific RH% and periodically weighed. The slope of the increasing weight over time, divided by exposure area A, gives the WVTR value of the films (5).

$$WVTR = \frac{slope\ (\frac{mass}{time})}{Area} \qquad (5)$$

The water vapour permeability (WVP) is closely related to the water vapor transmission rate (WVTR), but it further considers the film's thickness (t) and the moisture pressure difference (P) as a driving force (eq. 6). This pressure difference is established between the external environment (dependent on relative humidity, RH%) and the internal environment of the test cup (maintained at RH 0%).

$$WVP = \frac{WVTR * t}{P} \qquad (6)$$

***S1.5 Oil resistance ability of films (OAR %)*:** OAR% is particularly relevant for potential applications in high-fat content food. Film samples are fixed with parafilm on the top of a glass test tube containing sunflower oil and are placed on the filter paper upside down for 48 h. The filter paper is weighed after 48 h and OAR% calculation by eq. 7.

$$OAR\% = \frac{m_{48} - m_0}{m_0} * 100 \quad (7)$$

Where m_{48} is the mass of the filter after 48 h and m_0 is its starting mass.

S1.6 Mechanical properties: tensile strength (TS) and elongation at break (EB%): Most common mechanical properties present in the examined papers are TS (MPa) and EB%. TS gives information of the maximum force to be applied (F) to break a sample specimen with a given cross section area (A) (eq. 8).

$$TS = F/A \quad (8)$$

EB% is calculated by the maximum elongation that the sample specimen exhibits before breaking (eq. 9).

$$EB = \frac{l_b - l_0}{l_0} * 100 \quad (9)$$

Where l_b is the length of the specimen at maximum elongation and l_0 is the starting length. EB and TS are influenced by crosslinking, addition of plasticizers, reinforcement agents or additives. A careful balance between tensile strength (TS) and elongation at break (EB) is crucial, and the ideal compromise depends on the specific final application.

S1.7 Opacity and UV-barrier properties: The opacity can be measured quantitatively as reported in the literature [227] (eq. 10):

$$Opacity = \frac{Abs600}{t} \quad (10)$$

where *Abs600* is the absorbance of the film at 600 nm and *t* is the thickness in mm. Opacity strongly impacts the applicability of a given material for practical applications. Although an increase in opacity leads to higher light-barrier properties (avoiding undesired photochemical reaction) and positively affects the shelf-life of the product [228, 39, 159], on the other hand, low opacity makes the film more suitable and aesthetically attractive for the customer [71, 115]. Also in this case, a good compromise should be found, and the opacity level should be considered as a function of the final application.

UV barrier properties are measured at three different wavelengths (250 nm, 300 nm, 350 nm), representative of UVC, UVB, UVA. These data are particularly relevant to define the specific application, since such radiations generate undesired photochemical processes (cleavage of covalent bonds, generation of free radicals) responsible, for example, of the photodegradation of lipids in food.

S1.8 Antioxidant activity: Antioxidant activity is a key parameter for evaluating the effectiveness of CMC based films in acting as radical scavenger. In fact, radicals are in general very reactive species that can originate chain radical reactions with consequent product deterioration [157, 105, 165]. This parameter is usually evaluated by immersion of the film in a solution containing standard free radicals such as DPPH (2,2-diphenyl-1-picrylhydrazyl) and ABTS (2,2′-azino-bis(3-ethylbenzothiazoline-6-sulfonic acid) sodium salt. The decrease in radical concentration after a given period of time, measured by spectrophotometry gives the scavenger activity, calculated as reported in eq. 11:

$$Scavenger\ activity\ (\%) = \left(1 - \frac{A_1 - A_2}{A_0}\right) * 100$$

where A_0 is the absorbance of the initial radical, A_1 is the absorbance of sample (radical solution + film) and A_2 is the absorbance of a solution obtained replacing the radical species with an unreactive species (blank solution).

***S1.9 Antimicrobial properties*:** Antimicrobial properties of films are fundamental for packaging applications. Reducing or inhibiting microorganisms' growth, in fact, plays a key role in the shelf life of the product. These properties are usually evaluated by measuring the inhibition zone. Basically, circular film samples are placed in contact with agar inoculated with microorganisms. Then, the whole system is incubated and, if the sample exhibits antimicrobial properties, no microorganism growth will be observed around it [143, 144, 149]. The inhibition zone is measured using a calliper and expressed in millimetres (mm) by subtracting the sample radius from the total radius of the Petri dish. Typical microorganisms employed in the literature are *Staphylococcus Aureus* and *Escherichia Coli*.
